# Synthesis of Tetravalent Thio- and Selenogalactoside-Presenting Galactoclusters and Their Interactions with Bacterial Lectin PA-IL from *Pseudomonas aeruginosa*

**DOI:** 10.3390/molecules26030542

**Published:** 2021-01-21

**Authors:** Tünde Zita Illyés, Lenka Malinovská, Erzsébet Rőth, Boglárka Tóth, Bence Farkas, Marek Korsák, Michaela Wimmerová, Katalin E. Kövér, Magdolna Csávás

**Affiliations:** 1 Department of Organic Chemistry, University of Debrecen, Egyetem tér 1, H-4032 Debrecen, Hungary; illyestz@unideb.hu; 2 Central European Institute of Technology, Masaryk University, Kamenice 5, 625 00 Brno, Czech Republic; malinovska@mail.muni.cz (L.M.); korsakmarek@mail.muni.cz (M.K.); michaw@chemi.muni.cz (M.W.); 3 National Centre for Biomolecular Research, Faculty of Science, Masaryk University, Kotlářská 2, 611 37 Brno, Czech Republic; 4 Department of Pharmaceutical Chemistry, University of Debrecen, Egyetem tér 1, H-4032 Debrecen, Hungary; rothnej@gmail.com (E.R.); nyirabrany93@gmail.com (B.T.); 5 Department of Inorganic and Analytical Chemistry, University of Debrecen, Egyetem tér 1, H-4032 Debrecen, Hungary; farkasbence1104@gmail.com (B.F.); kover@science.unideb.hu (K.E.K.); 6 Department of Biochemistry, Faculty of Science, Masaryk University, Kotlářská 2, 611 37 Brno, Czech Republic; 7 Research Group for Molecular Recognition and Interaction, Hungarian Academy of Sciences, University of Debrecen, Egyetem tér 1, H-4032 Debrecen, Hungary

**Keywords:** selenoglycosides, galactoclusters, *Pseudomonas aeruginosa*, PA-IL lectin, multivalency

## Abstract

Synthesis of tetravalent thio- and selenogalactopyranoside-containing glycoclusters using azide-alkyne click strategy is presented. Prepared compounds are potential ligands of *Pseudomonas aeruginosa* lectin PA-IL. *P. aeruginosa* is an opportunistic human pathogen associated with cystic fibrosis, and PA-IL is one of its virulence factors. The interactions of PA-IL and tetravalent glycoconjugates were investigated using hemagglutination inhibition assay and compared with mono- and divalent galactosides (propargyl 1-thio- and 1-seleno-β-d-galactopyranoside, digalactosyl diselenide and digalactosyl disulfide). The lectin-carbohydrate interactions were also studied by saturation transfer difference NMR technique. Both thio- and seleno-tetravalent glycoconjugates were able to inhibit PA-IL significantly better than simple d-galactose or their intermediate compounds from the synthesis.

## 1. Introduction

Lectins from pathogenic organisms could be important virulence factors. These specific carbohydrate-binding proteins could be involved in the recognition and adhesion processes in host-pathogens interactions [1]. Consequently, carbohydrate-based inhibitors of lectins are promising potential therapeutics [2]. Lectins are usually multivalent oligomeric proteins, frequently displaying an avidity effect and increased affinity to complex glycosylated surfaces. Therefore, the multivalent inhibitors containing several carbohydrate residues are suitable for disrupting lectins’ binding to host cells or tissues [3]. The opportunistic pathogen *Pseudomonas aeruginosa* is a Gram-negative bacterium, causing chronic and potentially lethal lungs infections in immunocompromised humans, mainly patients suffering from cystic fibrosis (CF). It is the most widespread pulmonary pathogen associated with CF and significantly influences morbidity and mortality [4,5]. *P. aeruginosa* produces a tetrameric d-galactose-specific lectin named PA-IL (LecA) which is considered to be involved in adhesion, biofilm formation, cellular invasion and cytotoxicity [6,7,8,9,10].

**Figure 1:**
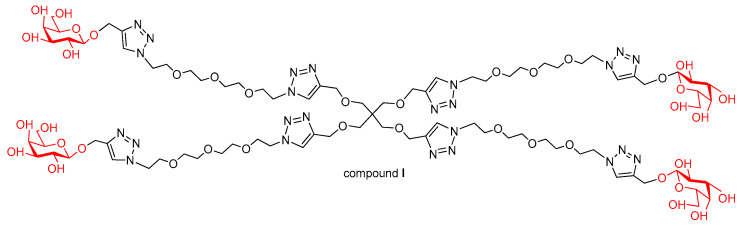
Lead compound (**I**) for anti-adhesion therapy of *Pseudomonas aeruginosa* infection.

Our previous works presented several potential inhibitors of various lectins of bacterial and fungal origin [11]. Recently, a tetravalent lead compound **I** for anti-adhesion therapy of *Pseudomonas aeruginosa* infections was developed (Figure 1) [12].

Our current aim is to synthesize the thio- and selenoglycoside analogues of the tetravalent lead-structure as well as their intermediate compounds to investigate their binding properties towards lectin PA-IL and the effect of sulfur and selenium on lectin binding. Several selenium-containing carbohydrates are known from the literature. They were synthesized for various purposes [13], exploiting the inherent potential of the selenium nucleus [14]. Selenoglycosides were used as glycosyl donors [15], for protein glycoconjugation in site-selective glycosylation by Se-S-mediated ligation [16], as enzyme inhibitors (*O*-GlcNAcase [17] and as novel glycosidase inhibitors [18]). Mono- and divalent selenogalactosides and diselenide digalactosides proved to be potential ligands to biomedically relevant galactophilic lectins [19]; non-glycosidically linked *Se*-containing pseudodisaccharides were also synthesized as BanLec and ConA lectin ligands [20]. Selenium-linked neoglycoconjugates, pseudodisaccharides [21], selenenylsulfide-linked glycopeptides and glycoproteins [22] were also prepared.  Moreover, the presence of a selenium nucleus provides an excellent opportunity for structural analysis of biomolecules by NMR and X-ray spectroscopy [14] and to study the lectin-carbohydrate interactions using sophisticated ^77^Se-NMR methods [23]. Selenium could be also potentially used as a trace for the selective detection of compounds in the biofluids [24]. A further advantage of the Se-interglycosidic linkage is its higher stability towards hydrolases [17,18].

## 2. Results and Discussion

### 2.1. Synthesis

PA-IL is known to recognize and interact with d-galactose-containing ligands specifically. In order to develop potential enzymatically stable ligands for this lectin, we have synthesized thio- and selenogalactoside-containing glycoconjugates based on the lead structure (Figure 1).

**Figure 2:**
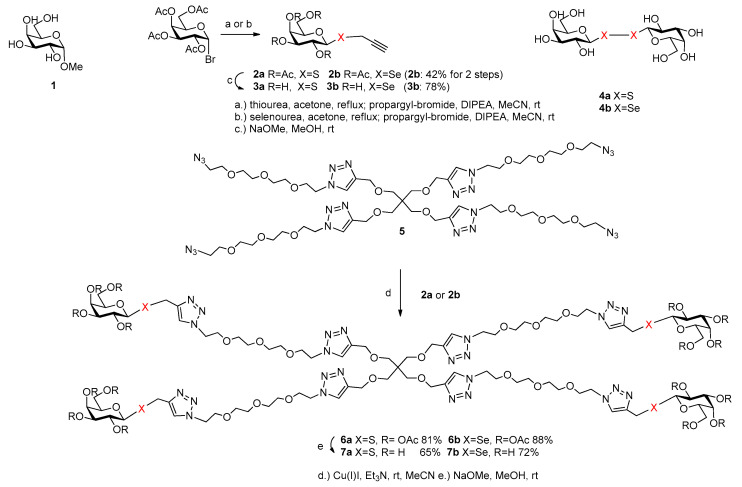
Synthesis of tetravalent thiogalactoside **7a** and selenogalactoside **7b**.

Methyl α-d-galactopyranoside **1** was used as standard, as a natural ligand of the lectin. For the synthesis of an oligovalent ligand by click-strategy [25], propargyl 2,3,4,6-tetra-*O*-acetyl-1-thio-β-d-galactopyranoside [26] (**2a**) and propargyl 2,3,4,6-tetra-*O*-acetyl-1-seleno-β-d-galactopyranoside (**2b**) were synthesized starting from peracetylated galactopyranoside bromide via thio- or selenouronium salt and propargylation (Figure 2). The deacetylated propargyl *S*-galactoside **3a [27]** and propargyl *Se*-galactoside **3b** were also suitable for investigations of their potential binding properties to the lectin PA-IL. Digalactosyl diselenide **4a** and digalactosyl disulfide **4b [19]** are known from the literature and were also suitable and available for binding studies as divalent galactoside ligands. The tetravalent glycoconjugates were built up by copper (I)-mediated azide-alkyne click reaction of alkyne **2a** or **2b** with azido-scaffold **5 [12]**. The acetylated thiogalactocluster **6a** and selenogalactocluster **6b** were isolated with a yield of 81% and 88%, respectively. The tetravalent galactoclusters **6a** and **6b** were deprotected by Zemplén-deacetylation method. Altogether, easy and efficient syntheses of the tetravalent thiogalactocluster **7a** and selenogalactocluster **7b** were achieved; moreover, monovalent thiogalactoside (**3a**) and selenogalactoside (**3b**), divalent selenogalactoside (**4a**) and thiogalactoside (**4b**) were also available for binding studies as potential ligands of galactose-specific PA-IL.

### 2.2. Inhibition of PA-IL with Thio- and Selenogalactosides

The inhibitory potential of tetravalent thio- and selenogalactosides as well as their intermediate compounds was investigated by hemagglutination inhibition assay with microscope detection [28]. The minimal inhibitory concentrations (MIC) of compounds were determined and their inhibitory potencies were calculated and semi-quantitatively evaluated by the comparison with simple monosaccharide d-galactose (standard). All tested compounds were able to inhibit hemagglutination caused by the lectin PA-IL (see Table 1 and Figure S3). The monovalent intermediates **3a** and **3b** (propargyl 1-thio- and 1-seleno-ß-d-galactopyranoside) showed eight times better inhibitory potency than d-galactose, possibly due to the additional interactions via their sidechains. Compounds **4a** and **4b** (digalactosyl disulfide and digalactosyl diselenide) displayed inhibitory potency 16 and 8, respectively. Although these compounds are theoretically divalent, they have no spacer and are not supposed to bind to the two binding sites simultaneously; therefore, the potencies comparable with monovalent compounds were expected. Both tetravalent compounds **7a** and **7b** were 256 times better inhibitors than d-galactose; taking into account the effect of several galactose units in the single compound (parameter β), they were 64 times better than d-galactose. These results are the same as the potency obtained for the lead structure **I**. The substitution of oxygen in the glycosidic linkage did not affect the inhibitory effect on lectin PA-IL.

### 2.3. STD-NMR Studies: Binding of Galactoside-Containing Ligands to PA-IL Lectin Characterized by ^1^H STD and Competition NMR Experiments

Ligand-based STD NMR experiments [29,30,31] were performed to support and further characterize the binding of β-d-galactoside-containing ligands to PA-IL. This technique is able to disclose the structural regions of the ligands that are involved in the binding. Moreover, further information on the interaction—such as binding site and relative affinity—can be obtained in competition experiments when suitable reference (natural) ligand is available.

**Figure 3:**
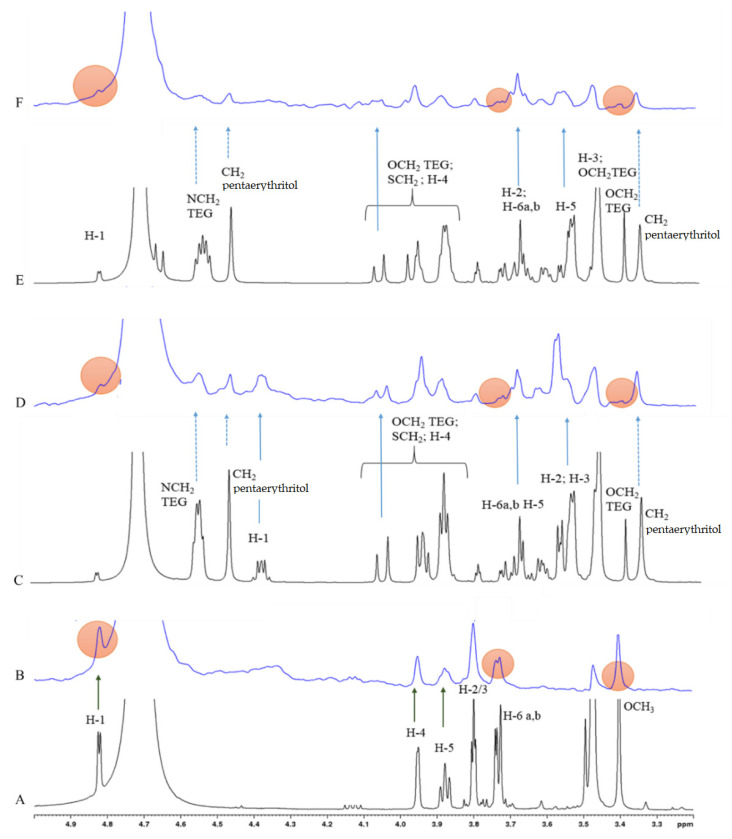
500 MHz ^1^H and STD NMR spectra of **1**, **7a** and **7b** in the presence of 10 μM PA-IL tetramer. (**A**,**B**) ^1^H and STD NMR spectra of **1**. (**C**,**D**) ^1^H and STD spectra of the 1:1 mixture of **1** and **7a**, (**E**,**F**) ^1^H and STD spectra of the 1:1 mixture of **1** and **7b**, respectively.

In the present study, Me α-d-Gal (**1**) served as a reference ligand and its binding to PA-IL was unambiguously confirmed with the STD NMR spectra shown in Figure 3B. All galactosyl ring protons and also the methyl protons of OCH_3_ group show similar STD effects (for resonance assignment see the ^1^H NMR spectrum in Figure 3A), suggesting that Me α-d-Gal being a small ligand may fit completely in the binding pocket of PA-IL.

In the succeeding competition experiments STD NMR spectra were recorded on samples containing both the natural (**1**) and one of the mono- or tetravalent ligands in a one-to-one molar ratio. The STD signals belonging to the well-resolved resonances (i.e., the ones separated from the resonances of **1** of tested compounds—marked by blue arrows in the STD spectra of Figure 3D,F, and also in Figure S2) confirm that all investigated mono- and tetravalent ligands bind to PA-IL. Moreover, the STD effects observed on the CH_2_ protons—marked by dotted blue arrows in Figure 3D,F—suggest that binding of compounds **7a** and **7b** to PA-IL involves certain hydrophobic contacts of the spacers in the formation of the complex.

Moreover, the STD signal attenuation of the reference ligand **1**—monitored on well-separated (non-overlapping) resonances of **1** and marked by filled red circles in Figure 3B,D,F—confirms that the particular ligands compete for the same (or partially overlapped) binding site of PA-IL. Considering the 1:1 ratio of the competing ligands in the sample, the substantial drop observed in the STD signal intensities suggest that the tetravalent ligands **7a** and **7b** show significantly higher affinity towards PA-IL than the natural ligand **1** used in the competition assay.

The STD NMR spectra of the monovalent ligands (**3a** and **3b**) show similar signal patterns (see Figure S2), confirming their binding to PA-IL. The attenuation of STD signals of **1** observed in the competition experiments, however, was significantly weaker, indicating lower affinity of the monovalent ligands towards PA-IL. It should be noted that the relative affinity order of the ligands assessed (qualitatively) in the competition NMR experiments is in accordance with the inhibition data given in Table 1.

In summary, tetravalent *S*- and *Se*-galactoclusters synthesized by click-chemistry were found to be suitable ligands of the lectin PA-IL in vitro, with significant, about 64 times better inhibitory activity than simple d-galactose. We can also conclude that enzymatically stable *S*- [32] and *Se*-interglycosidic linkages [17,18] do not influence the potency of ligands compared with the appropriate *O*-glycosides [12]. We could prove using STD-NMR techniques that the multivalent ligands compete with the natural ligand for the binding sites of the protein. In the future, multivalent selenoglycosides will provide a great opportunity to investigate the lectin-carbohydrate interactions in biologically relevant environments by highly sensitive and selective advanced ^77^Se-NMR methods. As d-galactose and l-fucose were applied for treatment of CF in an open clinical trial [33], novel *S*- and *Se*-galactoconjugates can be possible candidates in anti-adhesion therapy of cystic fibrosis as inhalational drugs.

## 3. Materials and Methods

### 3.1. General Methods

Optical rotations were measured at room temperature with a Perkin-Elmer 241 automatic polarimeter. TLC analysis was performed on Kieselgel 60 F_254_ (Merck) silica gel plates with visualization by immersing in a sulfuric-acid solution (5% in EtOH) followed by heating. Column chromatography was performed on silica gel 60 (Merck 0.063–0.200 mm) and flash column chromatography was performed on silica gel 60 (Merck 0.040–0.063 mm). Organic solutions were dried over MgSO_4_ and concentrated under vacuum. The ^1^H (500 MHz) and ^13^C NMR (125.76 MHz) spectra were recorded with Bruker Avance II 500 spectrometer (Bruker, Billerica, MA, USA). Chemical shifts are referenced to Me_4_Si or DSS (0.00 ppm for ^1^H) and to solvent signals (CDCl_3_: 77.00 ppm, CD_3_OD: 49.15 ppm for ^13^C). ESI-QTOF MS measurements were carried out on a maXis II UHR ESI-QTOF MS instrument (Bruker, Billerica, MA, USA), in positive ionization mode. The following parameters were applied for the electrospray ion source: capillary voltage: 3.6 kV; end plate offset: 500 V; nebulizer pressure: 0.5 bar; dry gas temperature: 200 °C and dry gas flow rate: 4.0 L/min. Constant background correction was applied for each spectrum, the background was recorded before each sample by injecting the blank sample matrix (solvent). Na-formate calibrant was injected after each sample, which enabled internal calibration during data evaluation. Mass spectra were recorded by otofControl version 4.1 (build: 3.5, Bruker, Billerica, MA, USA) and processed by Compass DataAnalysis version 4.4 (build: 200.55.2969) (Bruker, Billerica, MA, USA).

### 3.2. Synthesis

#### 3.2.1. Compound **2b**

Peracetylated galactopyranosyl bromide (1 g, 2.43 mmol) was dissolved in dry acetone (10 mL) and selenourea (300 mg, 2.43 mmol) was added then heated and stirred at reflux temperature for 1 h. When the TLC showed complete conversion of the starting material, it was evaporated, and the residue was dissolved in dry acetonitrile (10 mL). Propargyl bromide (80% solution in toluene, 1.2 mL, 2.8 mmol, 1.1 equiv.) and *N*,*N*-diisopropylethylamine (0.50 mL, 2.8 mmol) were added and stirred overnight at room temperature. The reaction mixture was evaporated, dissolved in ethyl acetate (50 mL), washed with distilled water (2 × 15 mL), dried over Na_2_SO_4_, filtered and evaporated. The crude product was purified by flash column chromatography (Merck, Darmstadt, Germany) (8:2 *n*-hexane:EtOAc) to give compound **2b** (482 mg, 42%) as a white powder. [α]^24^_D_—53.5 (*c* 0.32, CHCl_3_); R*_f_* 0.26 (7:3 *n*-hexane:EtOAc).

^1^H NMR (500 MHz, CDCl_3_) *δ* = 5.43 (d, *J* = 3.2 Hz, 1H, H-4), 5.28 (t, 1H, H-2), 5.05 (dd, *J* = 3.7 Hz, *J* = 9.9 Hz, 1H, H-3), 4.97 (d, *J* = 9.9 Hz, H, H-1), 4.17-4.04 (m, 2H, H-6a,b), 3.93 (m, 1H, H-5), 3.47 (dd, 1H, *J* = 15.4 Hz, *J* = 2.6 Hz, SeCH_2(A)_ propargyl), 3.26 (dd, 1H, *J* = 15.4 Hz, *J* = 2.6 Hz, SeCH_2(B)_ propargyl), 2.26 (t, *J* = 2.6 Hz, 1H, CH propargyl); 2.13, 2.04, 2.02, 1.96 (4 × s, 12H, 4 × CH_3_ acetyl), ppm; ^13^C-NMR (125 MHz, CDCl_3_): δ = 170.3, 170.1, 169.9, 169.7 (4C, 4 × CO acetyl), 79.8 (Cq propargyl), 77.7 (C-1), 75.6 (C-5), 71.7 (C-3), 71.5 (CH propargyl), 67.9 (C-2), 67.3 (C-4), 61.3 (C-6), 20.7, 20.6, 20.5 (4C, 4 × CH_3_ acetyl), 7.2 (SeCH_2_ propargyl) ppm.

ESI-HRMS: *m*/*z* calcd for C_17_H_22_NaO_9_Se [M+Na]^+^ 473.0327, found 473.0322.

#### 3.2.2. Compound **3b**

A catalytic amount of NaOMe (pH~9) was added to a stirred solution of ester **2b** (150 mg, 0.33 mmol) in dry MeOH (5 mL) and stirred overnight at room temperature. The reaction mixture was neutralized with Amberlite IR-120 H^+^ ion-exchange resin, filtered and evaporated; then, the crude product was purified by flash column chromatography (7:3 CH_2_Cl_2_:MeOH) to give compound **3b** (94 mg, 78%) as a colorless syrup. [α]^24^_D_—69.8 (*c* 0.21, MeOH); R*_f_* 0.34 (9:1 CH_2_Cl_2_:MeOH).

^1^H NMR (500 MHz, D_2_O) *δ* = 4.94 (d, *J* = 9.9 Hz, 1H, H-1), 3.98 (d, *J* = 2.1 Hz, 1H, H-4); 3.80–3.68 (overlapping signals, 4H, H-6a,b, H-2, H-5); 3.65 (dd, *J* = 2.1 Hz, *J* = 9.3 Hz, 1H, H-3); 3.59 (d, 1H, *J* 16.5 Hz, SeCH_2(A)_ propargyl), 3.49 (d, 1H, *J* 16.5 Hz, SeCH_2(B)_ propargyl) ppm; ^13^C-NMR (125 MHz, D_2_O): δ = 81.0 (Cpropargyl), 80.8 (C-1), 79.9 (C-5), 73.5 (C-3), 71.6 (CH propargyl), 69.8 (C-2), 68.5 (C-4), 60.7 (C-6), 6.2 (SeCH_2_ propargyl) ppm.

ESI-HRMS: *m*/*z* calcd for C_9_H_14_NaO_5_Se [M+Na]^+^ 304.9904, found 304.9900.

#### 3.2.3. Compound **6a**

Et_3_N (42 μL, 0.3 mmol, 4 equiv.) and Cu(I)I (5.7 mg, 0.03 mmol, 0.4 equiv.) were added to a stirred solution of propargyl 1-thiogalactoside peracetate **2a** (181 mg, 0.45 mmol, 6.0 equiv.) and azide scaffold **5** (95 mg, 0.075 mmol) in CH_3_CN (5 mL) under an argon atmosphere and stirred overnight at room temperature. The reaction mixture was evaporated, and the crude product was purified by flash column chromatography (95:5 CH_2_Cl_2_:MeOH) to give compound **6a** (176 mg, 81%) as a colorless syrup. [α]^24^_D_—28.8 (*c* 0.13, MeOH); R*_f_* 0.48 (95:5 CH_2_Cl_2_: MeOH).

^1^H NMR (500 MHz, CDCl_3_) *δ* = 7.75, 7.72 (2 × s, 8H, 8 × CH triazole), 5.41 (d, *J* = 2.8 Hz, 4H, 4 × H-4), 5.23 (dd, *J* = 8.0 Hz, *J* = 10.4 Hz, 4H, 4 × H-2), 5.03 (dd, *J* = 3.4 Hz, *J* = 10.4 Hz, 4H, 4 × H-3), 4.62 (d, *J* = 8.0 Hz, 4H, 4 × H-1), 4.50 (s, 8H, 4 × CH_2_ pentaerythritol), 4.40 (16H, 8 × NCH_2_ TEG), 4.15 (d, *J* 16.5, 4H, 4 × SCH_2(A)_), 4,10 (m, 8H, 4 × H-6a,b), 3.99-3.96 (m, 4H, 4 × H-5, 4H, 4× SCH_2(B)_), 3.70-3.65 (m, 20H, 10 × OCH_2_ TEG), 3.62–3.58 (m, 32H, 16 × OCH_2_ TEG), 3.48 (s, 8H, 4 × CH_2_ pentaerythritol), 2.15, 2.06, 1.98, 1.97 (4 × s, 48H, 16 × CH_3_ acetyl) ppm; ^13^C NMR (125 MHz, CDCl_3_) *δ* = 170.3, 170.2, 169.9, 169.6 (16C, 16 × CO acetyl), 145.0, 144.5 (8C, Cq triazole), 124.0, 123.5 (8C, CH triazole), 83.4 (4C, 4 × C-1), 74.3 (4C, 4 × C-5), 71.7 (4C, 4 × C-3), 70.4, 69.3 (28C, 24 × OCH_2_ TEG, 4 × CH_2_ pentaerythritol), 67.3 (8C, 4 × C-2, 4 × C-4), 64.6 (4C, 4 × CH_2_ pentaerythritol), 61.2 (4C, 4 × C-6), 50.5, 50.4 (8C, 8 × NCH_2_ TEG), 45.3 (1C, Cq pentaerythritol), 24.7 (4C, 4 × SCH_2_), 20.8, 20.7, 20.5 (16C, 16 × CH_3_ acetyl) ppm.

ESI-HRMS: *m*/*z* calcd for C_117_H_172_N_24_NaO_52_S_4_ [M+Na]^+^ 2896.0333, found: 2896.0350.

#### 3.2.4. Compound **6b**

Et_3_N (42 μL, 0.3 mmol, 4 equiv.) and Cu(I)I (5.7 mg, 0.03 mmol, 0.4 equiv.) were added to a stirred solution of propargyl 1-selenogalactoside peracetate **2b** (169 mg, 0.375 mmol, 5.0 equiv.) and azide scaffold **5** (95 mg, 0.075 mmol) in CH_3_CN (5 mL) under an argon atmosphere and stirred overnight at room temperature. The reaction mixture was evaporated, and the crude product was purified by flash column chromatography (95:5 CH_2_Cl_2_:MeOH) to give compound **6b** (121 mg, 88%) as a colorless syrup. [α]^24^_D_ + 23.1 (*c* 0.11, CHCl_3_); R*_f_* 0.38 (95:5 CH_2_Cl_2_: MeOH).

^1^H NMR (500 MHz, CDCl_3_) *δ* = 7.67, 7.60 (2 × s, 8H, 8 × CH triazole), 5.40 (d, *J* = 2.7 Hz, 4H, 4 × H-4), 5.25 (t, *J* = 10.1 Hz, 4H, 4 × H-2), 5.00 (dd, *J* = 3.2 Hz, *J* = 10.0 Hz, 4H, 4 × H-3), 4.84 (d, *J* = 10.0 Hz, 4H, 4 × H-1), 4.54–4.41 (m, 24 H, 4 × CH_2_ pentaerythritol, 8 × NCH_2_ TEG), 4.07 (m, 12H, 4H, 4 × SeCH_2(A),_ 4 × H-6a,b), 3.98–3.87 (m, 8H, 4 × H-5, 4H, 4 × SeCH_2(B)_), 3.82 (m, 20H, 10 × OCH_2_ TEG), 3.60–3.46 (m, 32H, 16 × OCH_2_ TEG), 3.41 (s, 8H, 4 × CH_2_ pentaerythritol), 2.15, 2.06, 1.98, 1.97 (4 × s, 48H, 16 × CH_3_ acetyl) ppm; ^13^C NMR (125 MHz, CDCl_3_) *δ* = 170.2, 170.0, 169.8, 169.6 (16C, 16 × CO acetyl), 145.0, 144.9 (8C, Cq triazole), 123.5, 122.8 (8C, CH triazole), 78.0 (4C, 4 × C-1), 75.4 (4C, 4 × C-5), 71.5 (4C, 4 × C-3), 70.3, 69.2 (28C, 24 × OCH_2_ TEG, 4 × CH_2_ pentaerythritol), 67.9 (4C, 4 × C-2), 67.2 (4C, 4 × C-4), 64.8 (4C, 4 × CH_2_ pentaerythritol), 61.1 (4C, 4 × C-6), 50.1, 49.9 (8C, 8 × NCH_2_ TEG), 45.2 (1C, Cq pentaerythritol), 20.7, 20.5, 20.4 (16C, 16 × CH_3_ acetyl), 15.6 (4C, 4 × SeCH_2_) ppm.

ESI-HRMS: *m/z* calcd for C_117_H_172_N_24_Na_2_O_52_Se_4_ [M+2Na]^2+^ 1555.4005, found 1555.3999 [M+2Na]^2+^.

#### 3.2.5. Compound **7a**

A catalytic amount of NaOMe (pH ~ 9) was added to a stirred solution of ester **6a** (115 mg, 0.4 mmol) in dry MeOH (5 mL) and stirred overnight at room temperature. The reaction mixture was neutralized with Amberlite IR-120 H^+^ ion-exchange resin, filtered and evaporated; then, the crude product was purified by flash column chromatography (7:3 CH_3_CN:H_2_O) to give compound **7a** (58 mg, 65%) as a colorless syrup. [α]^24^_D_ +21.5 (*c* 0.23, CHCl_3_); R*_f_* 0.23 (7:3 CH_3_CN:H_2_O).

^1^H NMR (500 MHz, D_2_O) *δ* = 7.95, 7.93 (2 × s, 8H, 8 × CH triazole), 4.60–4.51 (m, 16H, 8 × NCH_2_ TEG), 4.46 (d, 4H, 4 × CH_2_pentaerythritol), 4.38 (d, 1H, 4 × H-1, *J* = 9.8 Hz), 4.05 (d, *J* 16.5, 4H, 4 × SCH_2(A)_), 3.98–3.82 (m, 24H, 8 × OCH_2_ TEG, 4 × SCH_2(B)_, 4 × H-4), 3.75–3.67 (m, 8H, 4 × H-6a,b), 3.62 (m, 4H, 4 × H-5), 3.56–3.53 (m, 8H, 4 × H-3, 4 × H-2), 3.54 (m, 16H, 8 × OCH_2_ TEG), 3.48 (m, 16H, 8 × OCH_2_ TEG), 3.36 (m, 8H, 4 × CH_2_ pentaerythritol) ppm; ^13^C NMR (125 MHz, D_2_O) *δ* = 144.8, 144.1 (8C, Cq triazole), 125.1, 124.4 (8C, CH triazole), 85.0 (4C, 4 × C-1), 78.8 (4C, 4 × C-5), 73.8 (4C, 4 × C-3), 69.6 (12C, 12 × OCH_2_ TEG), 69.3 (12C, 12 × OCH_2_ TEG), 68.6 (8C, 4 × C-4, 4 × C-2), 68.2 (4C, 4 × CH_2_ pentaerythritol), 63.5 (4C, 4 × CH_2_ pentaerythritol), 60.9 (4C, 4 × C-6), 49.9 (16C, 16 × NCH_2_ TEG), 44.5 (1C, Cq pentaerythritol), 23.4 (4C, 4 × SCH_2_) ppm.

ESI-HRMS: *m*/*z* calcd for C_85_H_140_N_24_NaO_36_S_4_ [M+Na]^+^ 2223.8643, found 2223.8637.

#### 3.2.6. Compound **7b**

A catalytic amount of NaOMe (pH ~ 9) was added to a stirred solution of ester **6b** (100 mg, 0.26 mmol) in dry MeOH (5 mL) and stirred overnight at room temperature. The reaction mixture was neutralized with Amberlite IR-120 H^+^ ion-exchange resin, filtered and evaporated; then, the crude product was purified by flash column chromatography (7:3 CH_3_CN:H_2_O) to give compound **7b** (56 mg, 72%) as a colorless syrup. [α]^24^_D_—19.40 (*c* 0.53, H_2_O); R*_f_* 0.26 (7:3 CH_3_CN:H_2_O).

^1^H NMR (500 MHz, D_2_O + CD_3_OD) *δ* = 7.90, 7.89 (2 × s, 8H, 8 × CH triazole), 4.61 (d, *J* = 9.8 Hz 4H, H-1), 4.53–4.44 (m, 16H, 8 × NCH_2_ TEG), 4.41 (8H, 4 × CH_2_ pentaerythritol), 4.02 (d, *J* 16.5 Hz, 4H, 4 × SCH_2(A)_), 3.95–3.88 (m, 24H, 8 × OCH_2_ TEG, 4 × SeCH_2(B)_, 4 × H-4), 3.75–3.67 (m, 12H, 4 × H-2, 4 × H-6a,b), 3.57 (m, 4H, 4 × H-5), 3.54–3.46 (m, 20H, 4 × H-3, 8 × OCH_2_ TEG), 3.45–3.37 (m, 16H, 8 × OCH_2_ TEG), 3.31 (m, 8H, 4 × CH_2_ pentaerythritol) ppm; ^13^C NMR (125 MHz, D_2_O + CD_3_OD) *δ* = 147.0, 145.3 (8C, Cq triazole), 126.3, 125.3 (8C, CH triazole), 82.1 (4C, 4 × C-1), 81.4 (4C, 4 × C-5), 75.1 (4C, 4 × C-3), 71.4 (4C, 4 × C-2), 70.8, 70.6 (16C, 16 × OCH_2_ TEG), 70.0 (4C, 4 × C-4), 69.8 (8C, 8 × OCH_2_ TEG), 69.4 (4C, 4 × CH_2_ pentaerythritol), 64.8 (4C, 4 × CH_2_ pentaerythritol) 62.2 (4C, 4 × C-6), 51.1 (8C, 8 × NCH_2_ TEG), 45.9 (1C, Cq pentaerythritol), 15.4 (4C, 4 × SeCH_2_) ppm.

ESI-HRMS: *m*/*z* calcd for C_85_H_140_N_24_Na_2_O_36_Se_4_ [M+2Na]^2+^ 1219.3159, found 1219.3154 [M+2Na]^2+^.

### 3.3. Hemagglutination Inhibition Assay (HIA)

PA-IL was produced and purified as previously described [7]. The lectin was dissolved in the suitable buffer (20 mM Tris/HCl, 150 mM NaCl, 5 mM CaCl_2_, pH 7.5) to a concentration of 0.25 mg·mL^−1^. The lectin was mixed with synthetized galactosides and serially diluted in the buffer in a 5 µL:5 µL ratio. The final (working) concentration of the lectin was therefore 0.125 mg·mL^−1^. Then, a total volume of 10 µL of 20% papain-treated, azid-stabilized red blood cells B^−^ in the buffer was added, after which the mixture was thoroughly mixed and incubated for 5 min at room temperature. After incubation, the mixture was again mixed, transferred to a microscope slide and examined. The examination was conducted using the Levenhuk D2L NG Digital Microscope (Levenhuk, Tampa, FL, USA). Images were obtained with a Levenhuk D2L digital camera (Levenhuk, Tampa, FL, USA) using the software ToupView for Windows (Levenhuk, Tampa, FL, USA). The positive (experiment without an inhibitor) and negative control (experiment without the lectin) were prepared and processed in the same way using the appropriate volume of dissolving buffer instead of the omitted components. The minimal inhibitory concentration (MIC) of the inhibitor able to inhibit hemagglutination was determined and compared with the standard (d-galactose), and the potency of the inhibitor was calculated (MIC of the standard/MIC of the inhibitor).

### 3.4. ^1^H STD NMR Experiment

All NMR measurements were performed on a Bruker Avance II 500 spectrometer (Bruker, Billerica, MA, USA) operating at 500.13 MHz for ^1^H and equipped with 5-mm triple-resonance (txi) probe-head with *z*-axis gradients.

^1^H STD NMR spectra were recorded on samples dissolved in D_2_O (1M Tris-d_11_, 0.5 mM CaCl_2_, pH 7.5, T = 303 K) with the molar ratio of the ligand to PA-IL of about 100:1. The concentration of PA-IL tetramer was kept as low as ca. 10 μM to avoid aggregation upon addition of the ligand(s). For selective saturation of protein resonances, a train of band-selective E-BURP-1 (90°) shaped pulses of 50 ms each with a maximum B1 field strength of 75 Hz was employed yielding a total irradiation time of 3 s. For irradiation at aliphatic (CH_3_) region of PA-IL the E-BURP-1 pulses were applied at −0.3 ppm, while for recording the reference (off-resonance) spectrum, the irradiation frequency was set at −27 ppm. Off- and on-resonance data were recorded at alternate scans and the corresponding FIDs were collected in separate memories for subsequent processing and for the generation of STD spectra. Competition STD NMR experiments were performed following the experimental protocol as given above on samples containing two ligands (natural ligand and one of the mono- or multivalent ligands) and PA-IL lectin in 100:100:1 molar ratio. STD spectra were typically recorded with 2000–2400 transients to obtain a suitable signal-to-noise ratio for the analysis.

## Figures and Tables

**Table 1:** The MIC (minimal inhibitory concentration) values and potencies of tested inhibitors obtained for the inhibition of hemagglutination caused by PA-IL lectin from *Pseudomonas aeruginosa*.

Inhibitor	MIC	Potency ^2^	Valency	β ^3^
d-galactose ^1^	6.25 mM	1	1	1
Me α-d-Gal	1.562 mM	4	1	4
Compound **3a**	0.781 mM	8	1	8
Compound **3b**	0.781 mM	8	1	8
Compound **4a**	0.391 mM	16	2	8
Compound **4b**	0.781 mM	8	2	4
Compound **I** ^4^	24.41 µM	256	4	64
Compound **7a**	24.41 µM	256	4	64
Compound **7b**	24.41 µM	256	4	64

^1^ Standard, ^2^ MIC of standard/MIC of inhibitor, ^3^ Potency/Valency, ^4^ From ref. 12.

## Data Availability

The data presented in this study are available in Supplementary Material.

## Supplementary Materials

The following are available. Figure S1: NMR spectra of the new compounds. Figure S2: The STD NMR spectra of the monovalent ligand **1**, **3a** and **3b** in the presence of PA-IL. Figure S3: Influence of d-galactose, compounds **3a**, **3b**, **4a**, **4b**, **7a** and **7b** on hemagglutination caused by lectin PA-IL.

## Sample Availability

Samples of the compounds **2a**, **2b**, **3a**, **3b**, **4a**, **4b**, **7a** and **7b** are available from the authors.

## References

[1] Sharon N., Lis H. (1998). Lectins: Carbohydrate-specific proteins that mediate cellular recognition. Chem. Rev..

[2] Sharon N. (2006). Carbohydrates as future anti-adhesion drugs for infectious diseases. Biochim. Biophys. Acta.

[3] Cecioni S., Imberty A., Vidal S. (2015). Glycomimetics versus Multivalent Glycoconjugates for the Design of High Affinity Lectin Ligands. Chem. Rev..

[4] Folkesson A., Jelsbak L., Yang L., Johansen H.K., Ciofu O., Høiby N., Molin S. (2012). Adaptation of *Pseudomonas aeruginosa* to the cystic fibrosis airway: An evolutionary perspective. Nat. Rev. Microbiol..

[5] Bhagirath A.Y., Li Y., Somayajula D., Dadashi M., Badr S., Duan K. (2016). Cystic fibrosis lung environment and *Pseudomonas aeruginosa* infection. BMC Pulm. Med..

[6] Cioci G., Mitchell E.P., Gautier C., Wimmerová M., Sudakevitz D., Pérez S., Gilboa-Garber N., Imberty A. (2003). Structural basis of calcium and galactose recognition by the lectin PA-IL of *Pseudomonas aeruginosa*. FEBS Lett..

[7] Chemani C., Imberty A., de Bentzmann S., Pierre M., Wimmerová M., Guery B.P., Faure K. (2009). Role of LecA and LecB lectins in *Pseudomonas aeruginosa*-induced lung injury and effect of carbohydrate ligands. Infect. Immun..

[8] Diggle S.P., Stacey R.E., Dodd C., Cámara M., Williams P., Winzer K. (2006). The galactophilic lectin, LecA, contributes to biofilm development in *Pseudomonas aeruginosa*. Environ. Microbiol..

[9] Novoa A., Eierhoff T., Topin J., Varrot A., Barluenga S., Imberty A., Römer W., Winssinger N. (2014). A LecA Ligand Identified from a Galactoside-Conjugate Array Inhibits Host Cell Invasion by *Pseudomonas aeruginosa*. Angew. Chem. Int. Ed..

[10] Bajolet-Laudinat O., Bentzmann S.G.-D., Tournier J.M., Madoulet C., Plotkowski M.C., Chippaux C., Puchelle E. (1994). Cytotoxicity of *Pseudomonas aeruginosa* internal lectin PA-I to respiratory epithelial cells in primary culture. Infect. Immun..

[11] Thai L.S., Malinovská L., Vašková M., Mező E., Kelemen V., Borbás A., Hodek P., Wimmerová M., Csávás M. (2019). Investigation of the Binding Affinity of a Broad Array of L-Fucosides with Six Fucose-Specific Lectins of Bacterial and Fungal Origin. Molecules.

[12] Malinovská L., Thai L.S., Herczeg M., Vašková M., Houser J., Fujdiarová E., Komárek J., Hodek P., Borbás A., Wimmerová M. (2019). Synthesis of β-D-galactopyranoside-presenting glycoclusters, investigation of their interactions with *Pseudomonas aeruginosa* lectin A (PA-IL) and evaluation of their anti-adhesion potential. Biomolecules.

[13] Mangiavacchi F., Dias I.F.C., Di Lorenzo I., Grzes P., Palomba M., Rosati O., Bagnoli L., Marini F., Santi C., Lenardao E.J. (2020). Sweet Selenium: Synthesis and Properties of Selenium-Containing Sugars and Derivatives. Pharmaceuticals.

[14] Suzuki T., Makyio H., Ando H., Komura N., Menjo M., Yamada Y., Imamura A., Ishida H., Wakatsuki S., Kato R. (2014). Expanded potential of seleno-carbohydrates as a molecular tool for X-ray structural determination of a carbohydrate–protein complex with single/multi-wavelength anomalous dispersion phasing. Bioorg. Med. Chem..

[15] Valerio S., Iadonisi A., Adinolfi M., Ravidá A. (2007). Novel Approaches for the Synthesis and Activation of Thio- and Selenoglycoside Donors. J. Org. Chem..

[16] Gamblin D.P., Garnier P., Van Kasteren S., Oldham N.J., Fairbanks A.J., Davis B.G. (2004). Glyco-SeS: Selenenylsulfide-Mediated Protein Glycoconjugation—A New Strategy in Post-Translational Modification. Angew. Chem. Int. Ed..

[17] Kim E.J., Love N.C., Darout E., Abdo M., Rempel B., Withers S.G., Rablen P.R., Hanover J.A., Knapp S. (2010). OGA inhibition by GlcNAc-selenazoline. Bioorg. Med. Chem..

[18] Mehta S., Andrews J.S., Svensson B., Pinto B.M. (1995). Synthesis and Enzymic Activity of Novel Glycosidase Inhibitors Containing Sulfur and Selenium. J. Am. Chem. Soc..

[19] André S., Kövér K.E., Gabius H.-J., Szilágyi L. (2015). Thio- and selenoglycosides as ligands for biomedically relevant lectins: Valency–activity correlations for benzene-based dithiogalactoside clusters and first assessment for (di)selenodigalactosides. Bioorg. Med. Chem. Lett..

[20] Cumpstey I., Ramstadius C., Akhtar T., Goldstein I.J., Winter H.C. (2010). Non-Glycosidically Linked Pseudodisaccharides: Thioethers, Sulfoxides, Sulfones, Ethers, Selenoethers, and Their Binding to Lectins. Eur. J. Org. Chem..

[21] Affeldt R.F., Braga H.C., Baldassari L.L., Luedtke D.S. (2012). Synthesis of selenium-linked neoglycoconjugates and pseudodisaccharides. Tetrahedron.

[22] Boutureira O., Bernardes G.J.L., Fernández-González M., Anthony D.C., Davis B.G. (2011). Selenenylsulfide-Linked Homogeneous Glycopeptides and Glycoproteins: Synthesis of Human “Hepatic Se Metabolite A”. Angew. Chem. Int. Ed..

[23] Raics M., Timári I., Diercks T., Szilágyi L., Gabius H.-J., Kövér K.E. (2019). Selenoglycosides as Lectin Ligands: ^77^Se-Edited CPMG-HSQMBC NMR Spectroscopy To Monitor Biomedically Relevant Interactions. ChemBioChem.

[24] Llamas I., Boutureira O., Claridge T.D.W., Davis B.G. (2015). Glycosyldiselenides as lectin ligands detectable by NMR in biofluids. Chem. Commun..

[25] Dondoni A. (2007). Triazole: The Keystone in Glycosylated Molecular Architectures Constructed by a Click Reaction. Chem. Asian J..

[26] Ziegler T., Pietrzik N., Schips C. (2008). Efficient Synthesis of Glycosylated Asparaginic Acid Building Blocks via Click Chemistry. Synthesis.

[27] Giguere D., Bonin M.-A., Cloutier P., Patnam R., St-Pierre C., Sato S., Roy R. (2008). Synthesis of stable and selective inhibitors of human galectins-1 and -3. Bioorg. Med. Chem..

[28] Adamová L., Malinovská L., Wimmerová M. (2014). New sensitive detection method for lectin hemagglutination using microscopy. Microsc. Res. Tech..

[29] Mayer M., Meyer B. (1999). Characterization of Ligand Binding by Saturation Transfer Difference NMR Spectroscopy. Angew. Chem. Int. Ed..

[30] Mayer M., Meyer B. (2001). Group Epitope Mapping by Saturation Transfer Difference NMR To Identify Segments of a Ligand in Direct Contact with a Protein Receptor. J. Am. Chem. Soc..

[31] Groves P., Kövér K.E., André S., Bandorowicz-Pikuła J., Batta G., Bruix M., Buchet R., Canales Á., Cañada F.J., Gabius H.-J. (2007). Temperature dependence of ligand-protein complex formation as reflected by saturation transfer difference NMR experiments. J. Magn. Reson. Chem..

[32] Driguez H. (1997). Thiooligosaccharides in glycobiology. Glycoscience Synthesis of Substrate Analogs and Mimetics.

[33] Hauber H.-P., Schulz M., Pforte A., Mack D., Zabel P., Schumacher U. (2008). Inhalation with Fucose and Galactose for Treatment of *Pseudomonas Aeruginosa* in Cystic Fibrosis Patients. Int. J. Med. Sci..

