# Supplementary material for: Synthesis of Tetravalent Thio- and Selenogalactoside-Presenting Galactoclusters and Their Interactions with Bacterial Lectin PA-IL from Pseudomonas aeruginosa
Source: Molecules. 2021 Jan 21;26(3):542. doi: 10.3390/molecules26030542 (PMC7865631; doi:10.3390/molecules26030542)
Supplement: Supplementary file 1 [file molecules-26-00542-s001.pdf]

Article

Synthesis of Tetravalent Thio- and Selenogalactoside-Presenting Galactoclusters and Their Interactions with Bacterial Lectin PA-IL from *Pseudomonas aeruginosa*

Tünde Zita Illyés [1], Lenka Malinovská [2,3], Erzsébet Rőth [4], Boglárka Tóth [4], Bence Farkas [7], Marek Korsák [2,3], Michaela Wimmerová [2,3,5], Katalin E. Kövér [6,7] and Magdolna Csávás [4,6,*]

1 Department of Organic Chemistry, University of Debrecen, Egyetem tér 1, H-4032 Debrecen, Hungary; illyestz@unideb.hu
2 Central European Institute of Technology, Masaryk University, Kamenice 5, 625 00 Brno, Czech Republic; malinovska@mail.muni.cz (L.M.); korsakmarek@mail.muni.cz (M.K.); michaw@chemi.muni.cz (M.W.)
3 National Centre for Biomolecular Research, Faculty of Science, Masaryk University, Kotlářská 2, 611 37 Brno, Czech Republic
4 Department of Pharmaceutical Chemistry, University of Debrecen, Egyetem tér 1, H-4032 Debrecen, Hungary; rothnej@gmail.com (E.R.); nyirabrany93@gmail.com (B.T.)
5 Department of Biochemistry, Faculty of Science, Masaryk University, Kotlářská 2, 611 37 Brno, Czech Republic
6 Research Group for Molecular Recognition and Interaction, Hungarian Academy of Sciences, University of Debrecen, Egyetem tér 1, H-4032 Debrecen, Hungary; kover@science.unideb.hu
7 Department of Inorganic and Analytical Chemistry, University of Debrecen, Egyetem tér 1, H-4032 Debrecen, Hungary; farkasbence1104@gmail.com
* Correspondence: csavas.magdolna@science.unideb.hu

Abstract: Synthesis of tetravalent thio- and selenogalactopyranoside-containing glycoclusters using azide-alkyne click strategy is presented. Prepared compounds are potential ligands of *Pseudomonas aeruginosa* lectin PA-IL. *P. aeruginosa* is an opportunistic human pathogen associated with cystic fibrosis, and PA-IL is one of its virulence factors. The interactions of PA-IL and tetravalent glycoconjugates were investigated using hemagglutination inhibition assay and compared with mono- and divalent galactosides (propargyl 1-thio- and 1-seleno-β-D-galactopyranoside, digalactosyl diselenide and digalactosyl disulfide). The lectin-carbohydrate interactions were also studied by STD-NMR technique. Both thio- and seleno-tetravalent glycoconjugates were able to inhibit PA-IL significantly better than simple D-galactose or their intermediate compounds from the synthesis.

Keywords: selenoglycosides; galactoclusters; *Pseudomonas auruginosa*; PA-IL lectin; multivalency

Citation: Illyés, T.Z.; Malinovská, L.; Rőth, E.; Tóth, B.; Farkas, B.; Korsák, M.; Wimmerová, M.; Kövér, K.E.; Csávás, M. Synthesis of Tetravalent Thio- and Selenogalactoside-Presenting Galactoclusters and Their Interactions with Bacterial Lectin PA-IL from *Pseudomonas aeruginosa*. *Molecules* **2021**, *26*, 542. https://doi.org/10.3390/molecules26030542

Academic Editor: George Grant

Received: 15 December 2020
Accepted: 18 January 2021
Published: 21 January 2021

Publisher's Note: MDPI stays neutral with regard to jurisdictional claims in published maps and institutional affiliations.

Copyright: © 2021 by the authors. Licensee MDPI, Basel, Switzerland. This article is an open access article distributed under the terms and conditions of the Creative Commons Attribution (CC BY) license (http://creativecommons.org/licenses/by/4.0/).

Figure S1. NMR spectra of compounds: **2b, 3b, 6a, 7a, 6b, 7b**

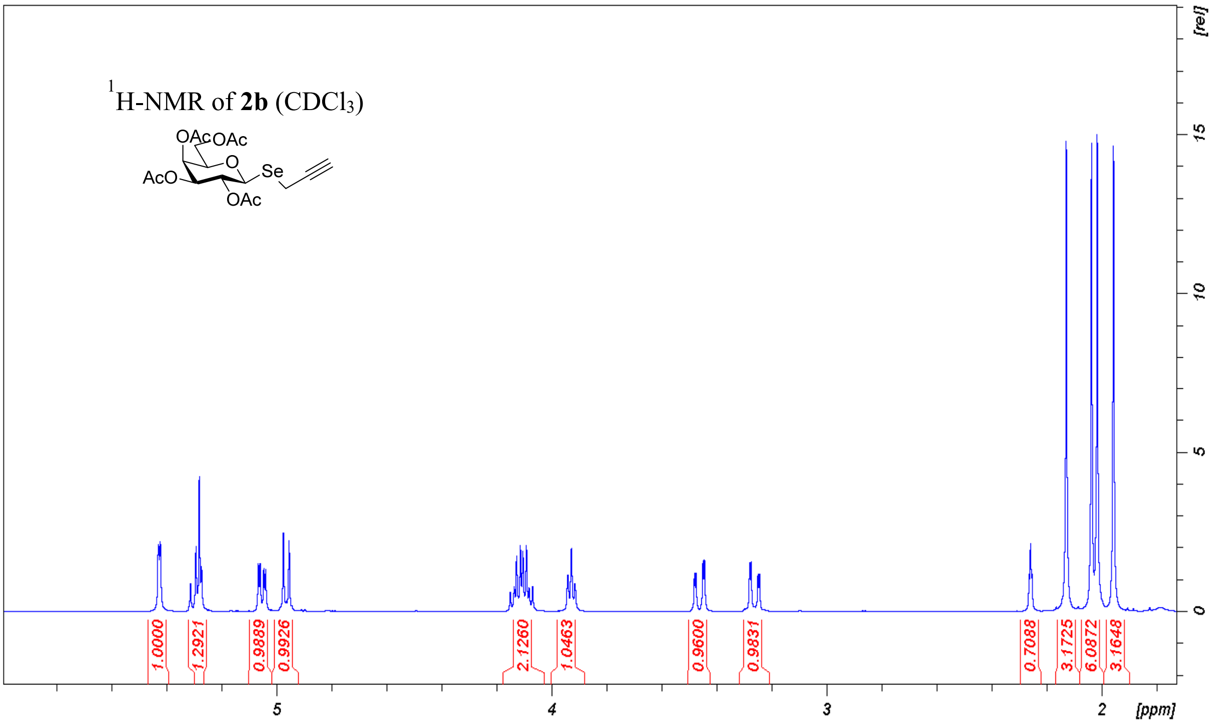

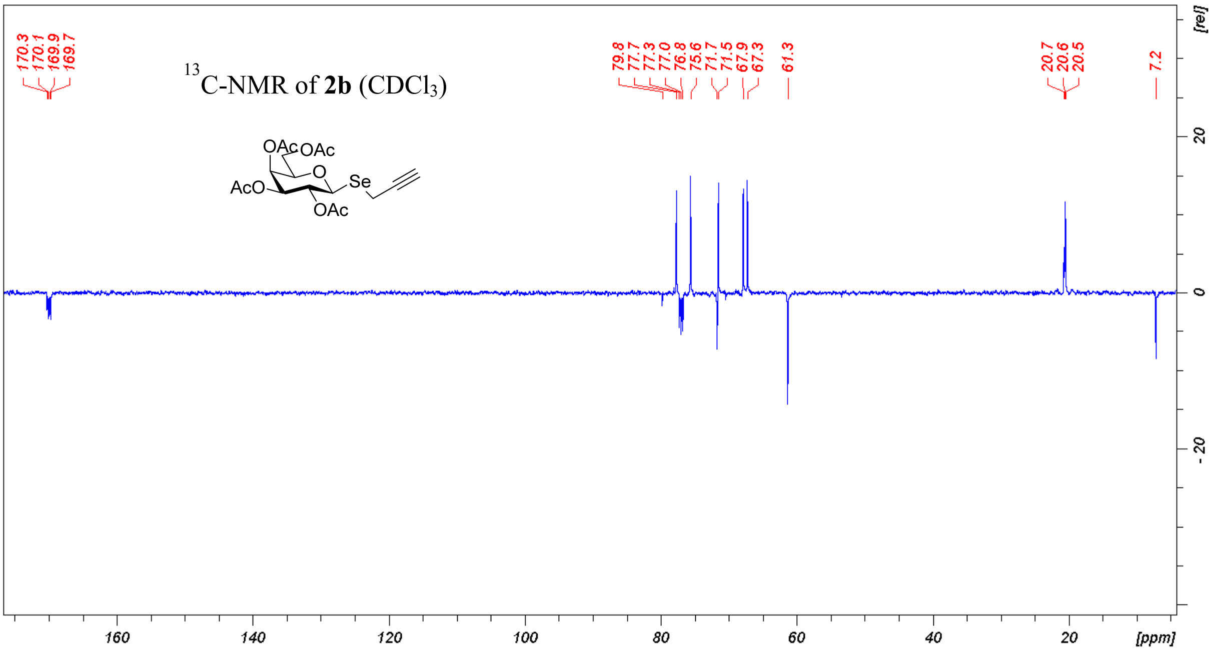

Figure S1. cont.

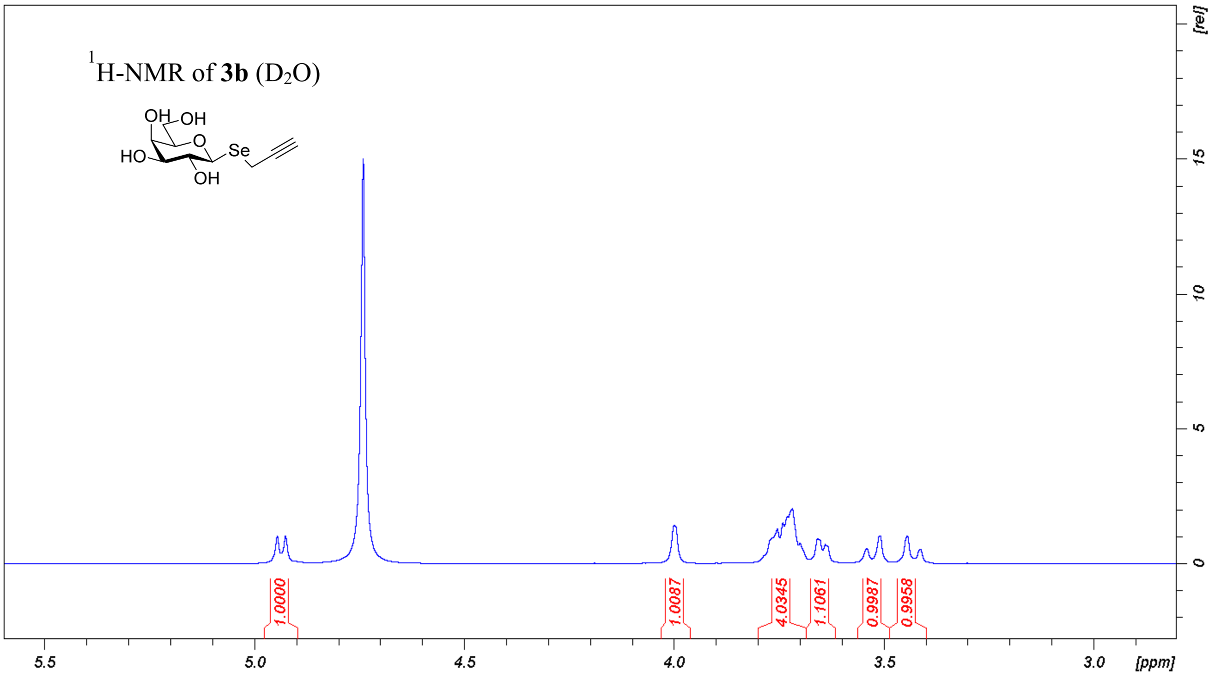

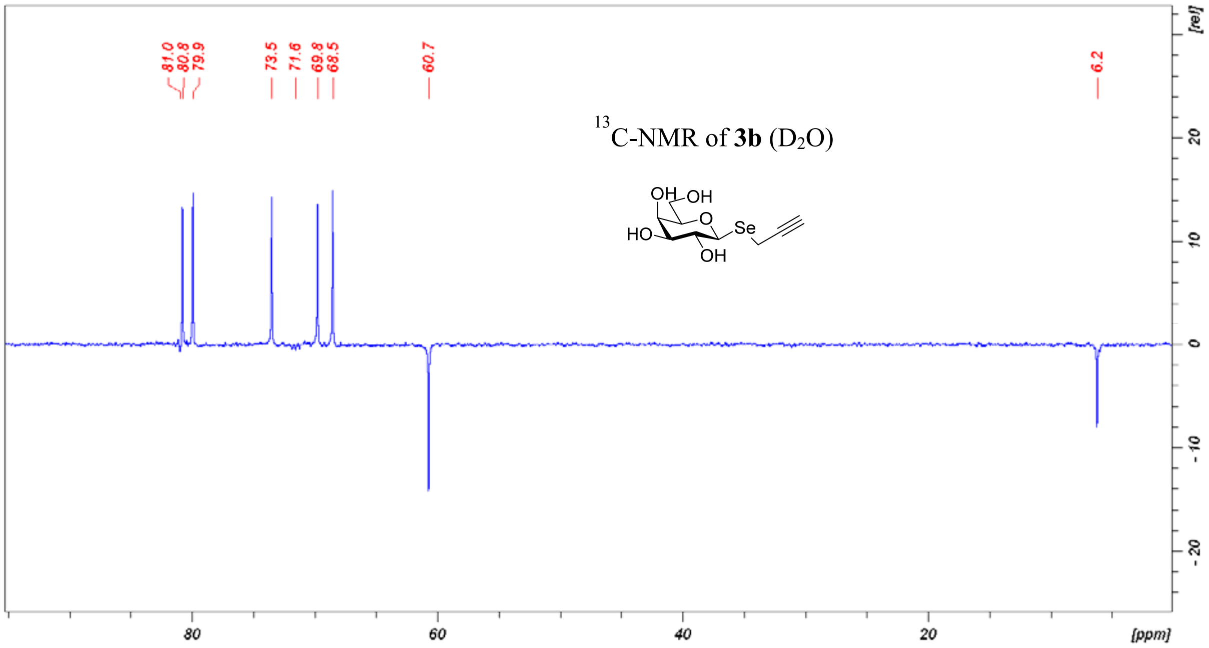

Figure S1. cont.

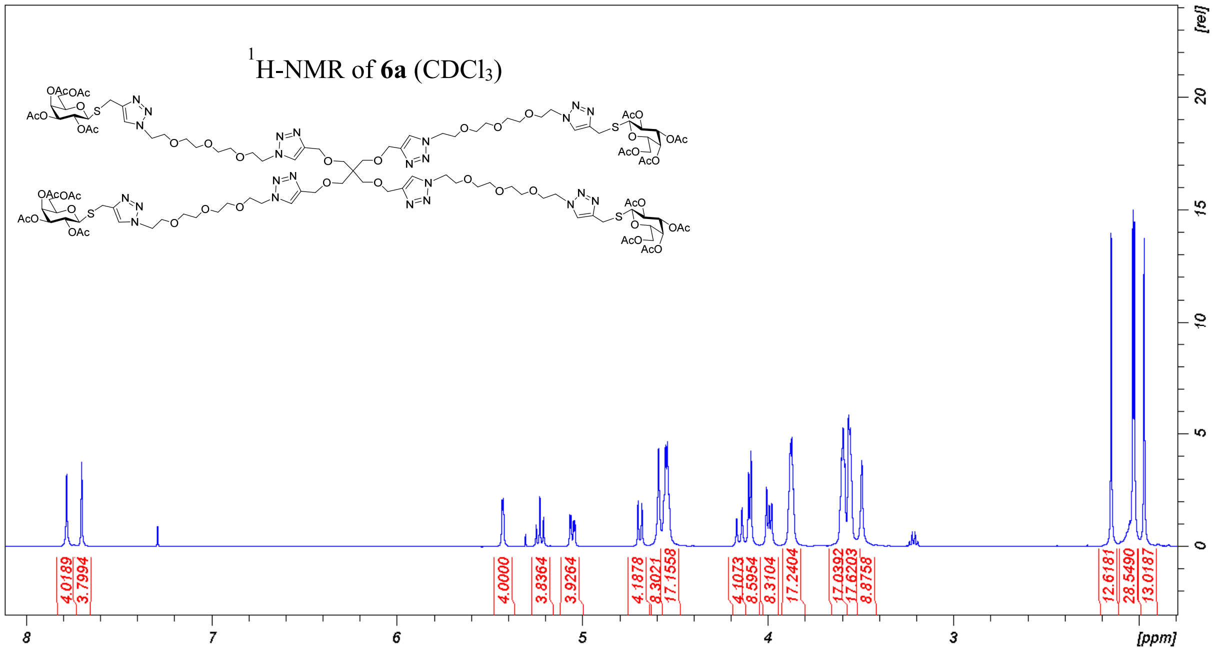

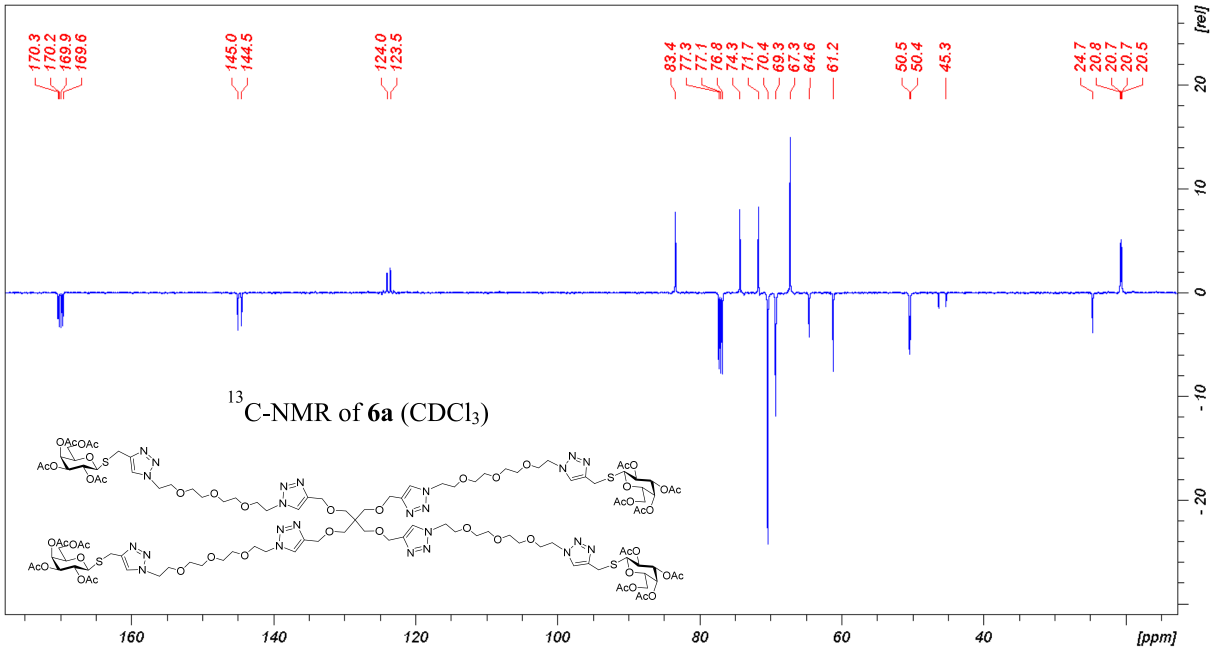

Figure S1. cont.

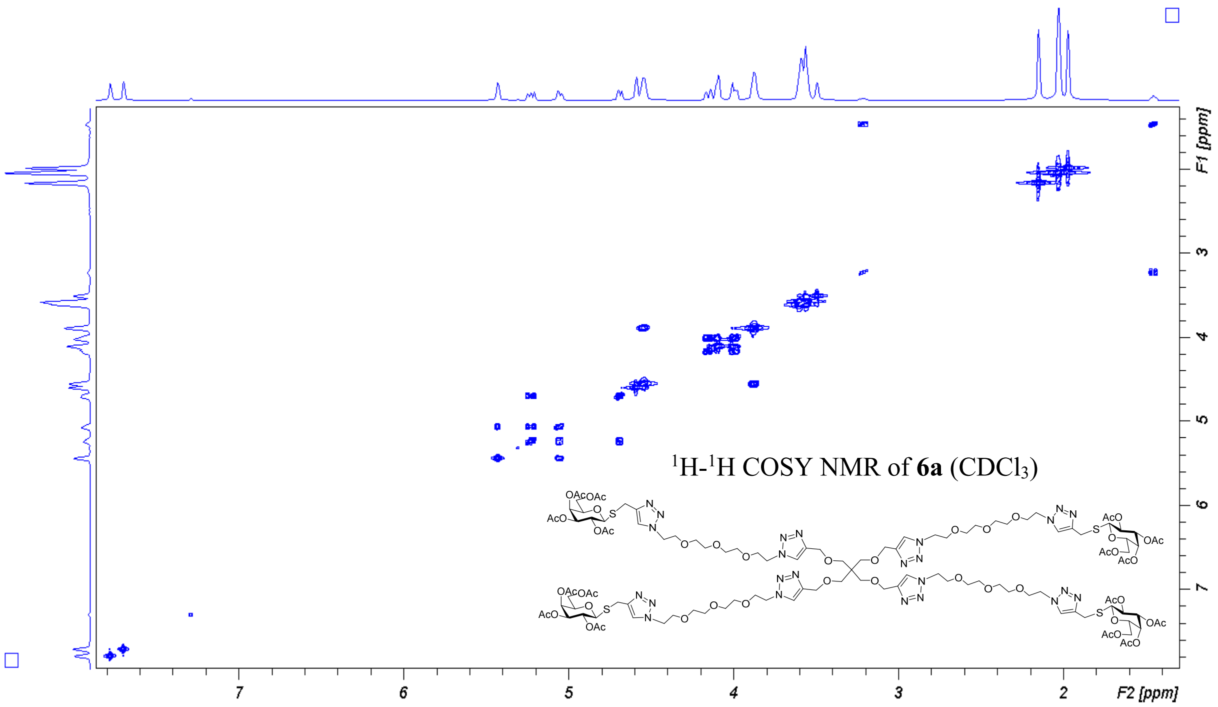

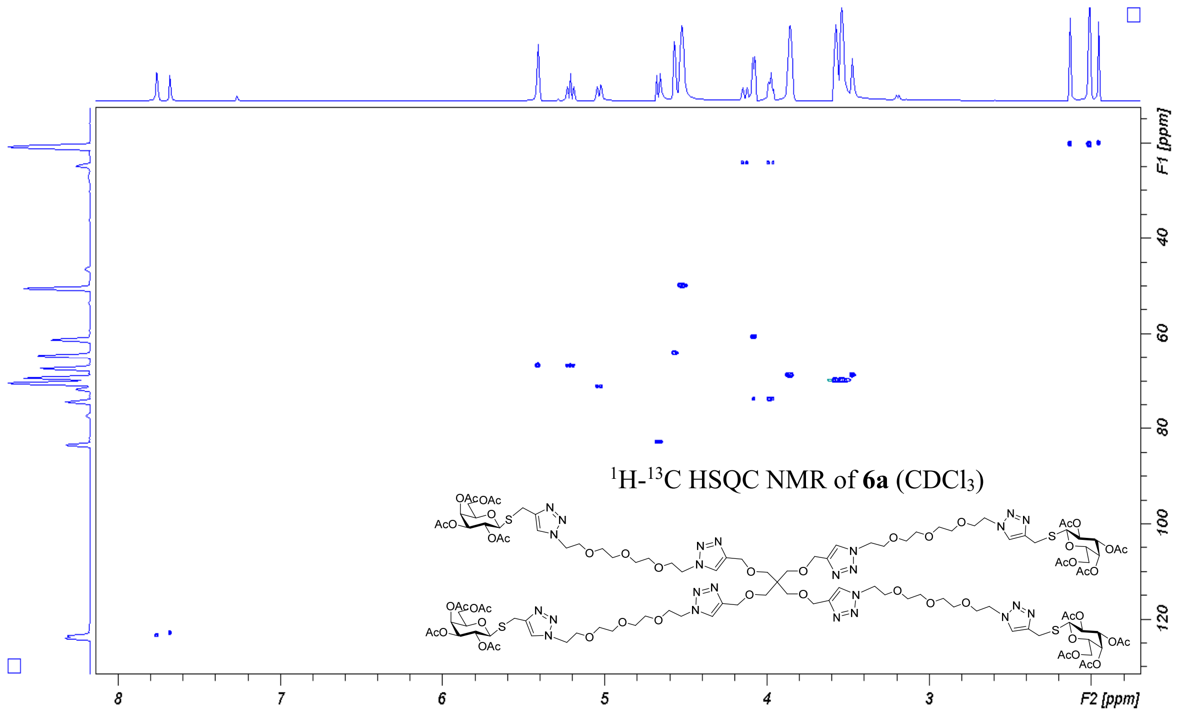

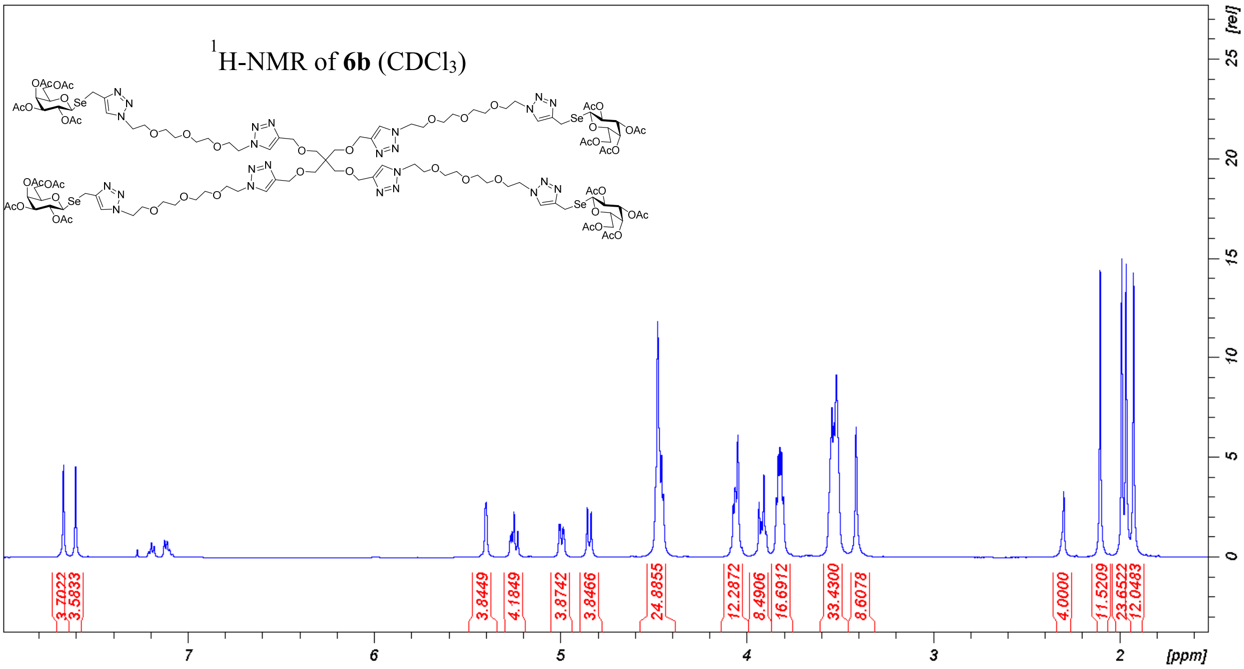

1H-NMR of 6b (CDCl3)
3.7022
3.5833
3.8449
4.1849
3.8742
3.8466
24.8855
12.2872
8.4906
16.6912
33.4300
8.6078
4.0000
11.5209
23.6522
12.0483
[ppm]
[rel]

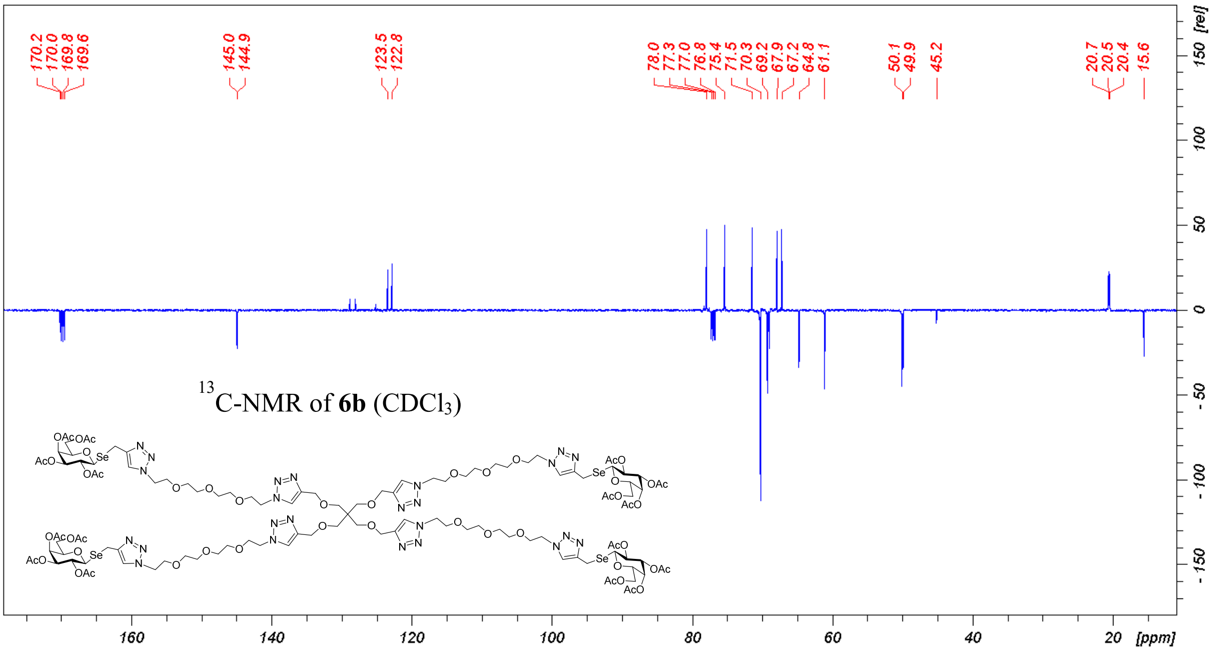

13C-NMR of 6b (CDCl3)
170.2
170.0
169.8
169.6
145.0
144.9
123.5
122.8
78.0
77.3
77.0
76.8
75.4
71.5
70.3
69.2
67.9
67.2
64.8
61.1
50.1
49.9
45.2
20.7
20.5
20.4
15.6
[ppm]
[rel]

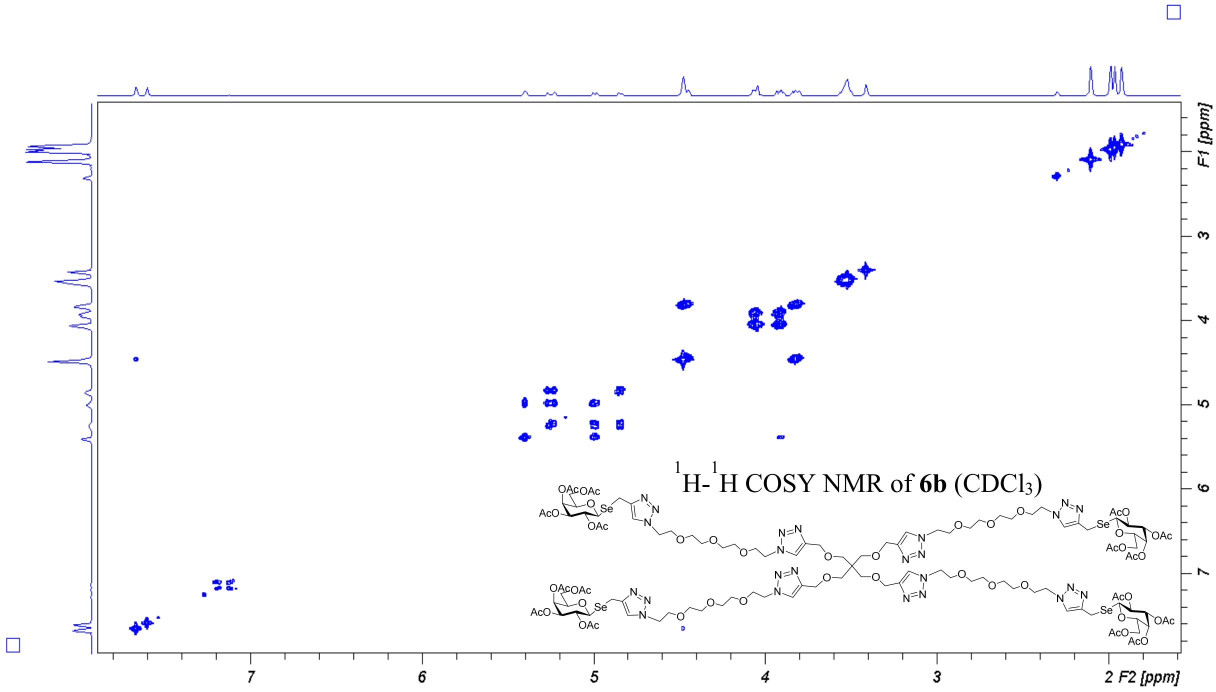

^{1}H-^{1}H COSY NMR of **6b** ($CDCl_3$)

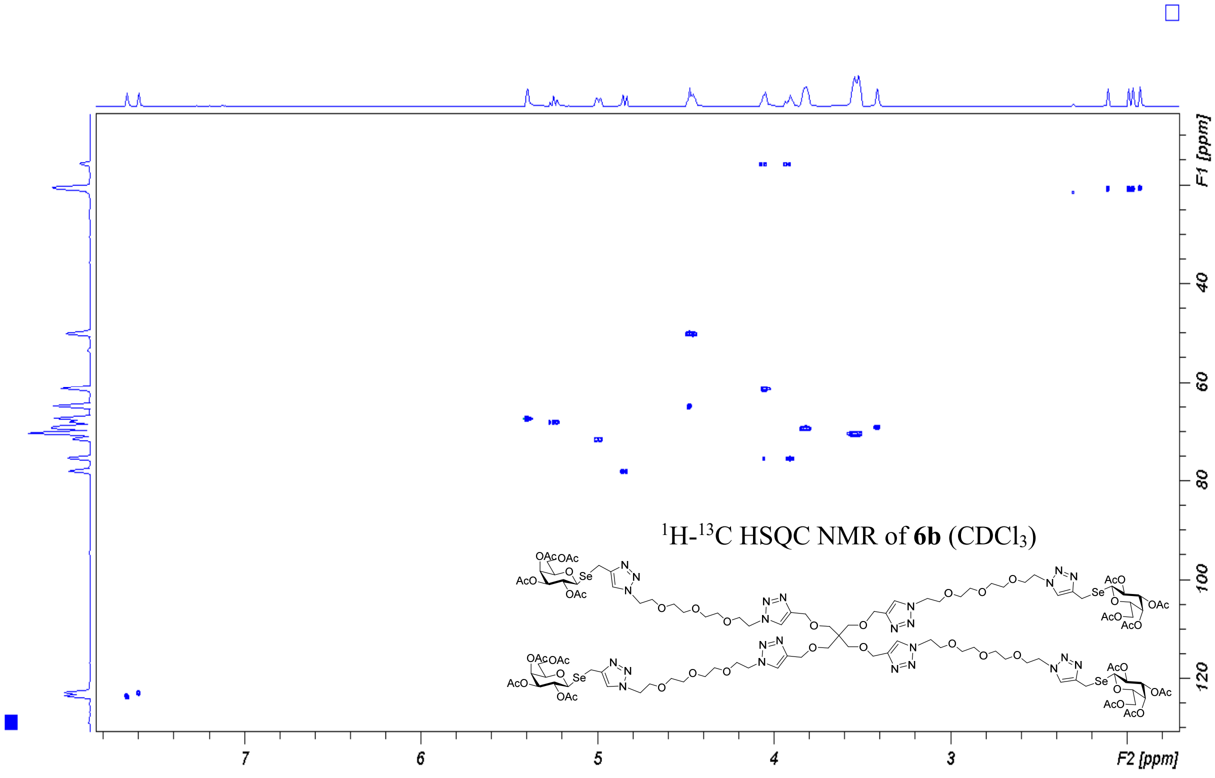

^{1}H-^{13}C HSQC NMR of **6b** ($CDCl_3$)

Figure S1. cont.

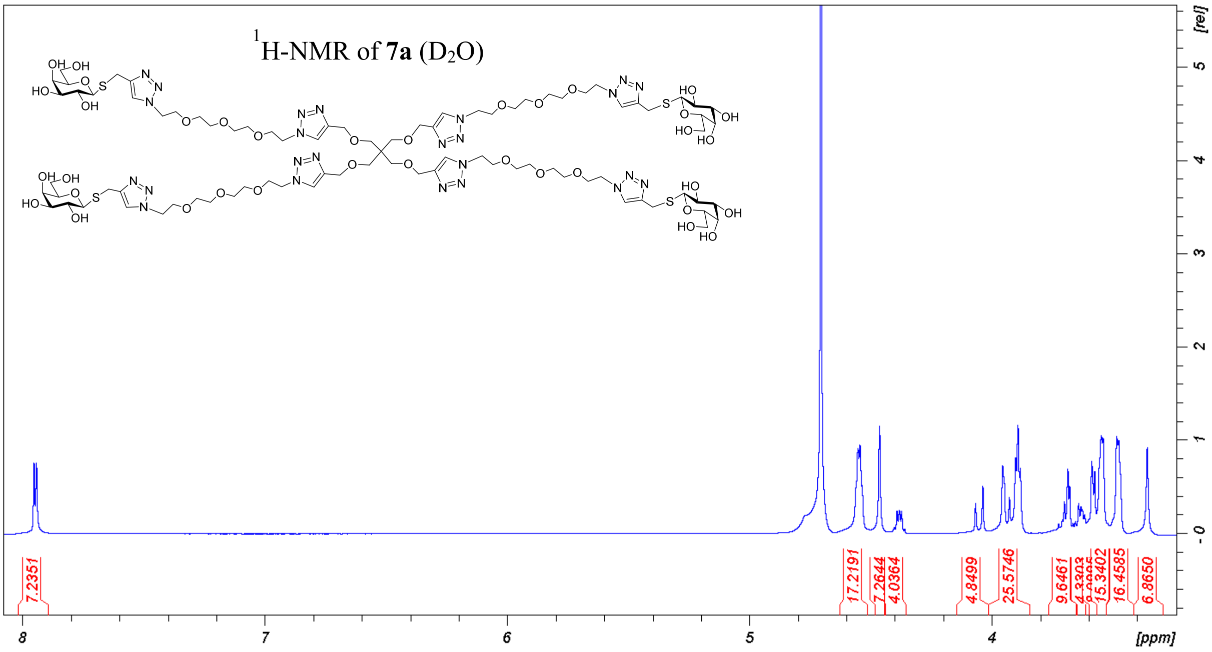

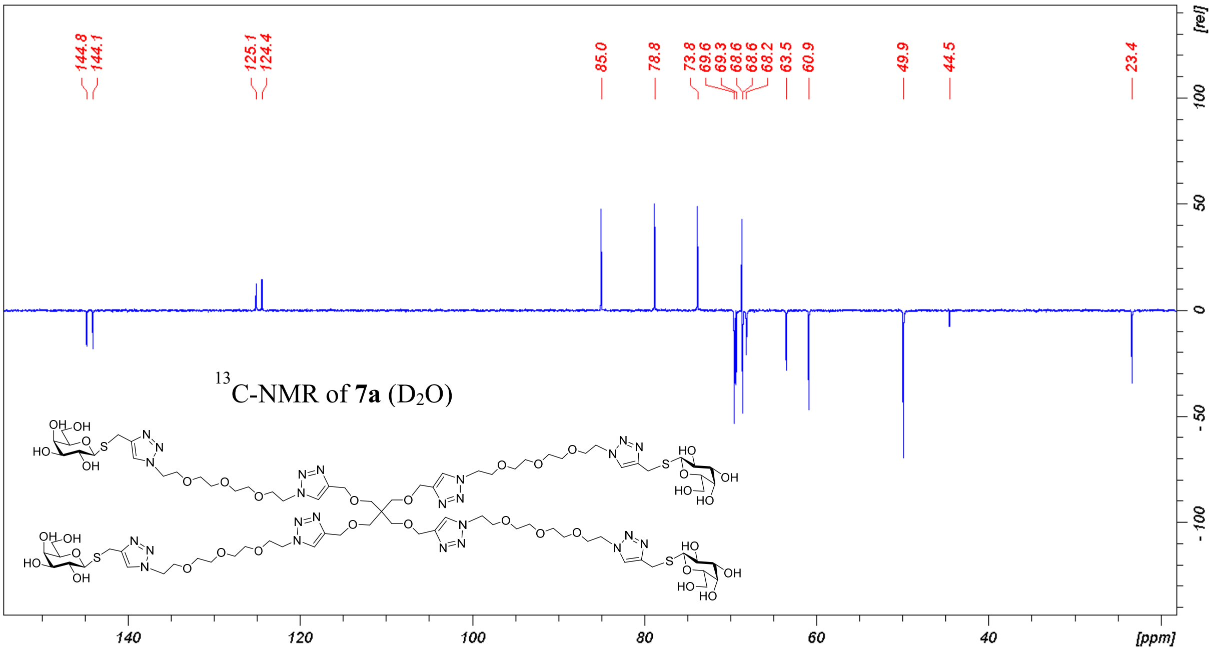

Figure S1. cont.

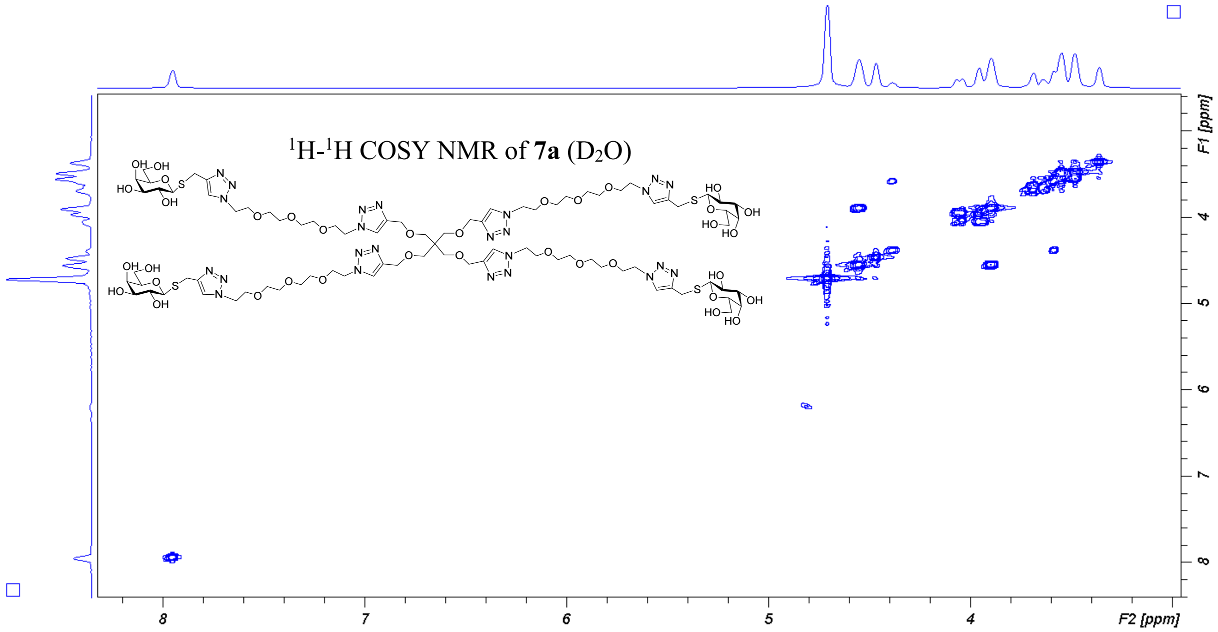

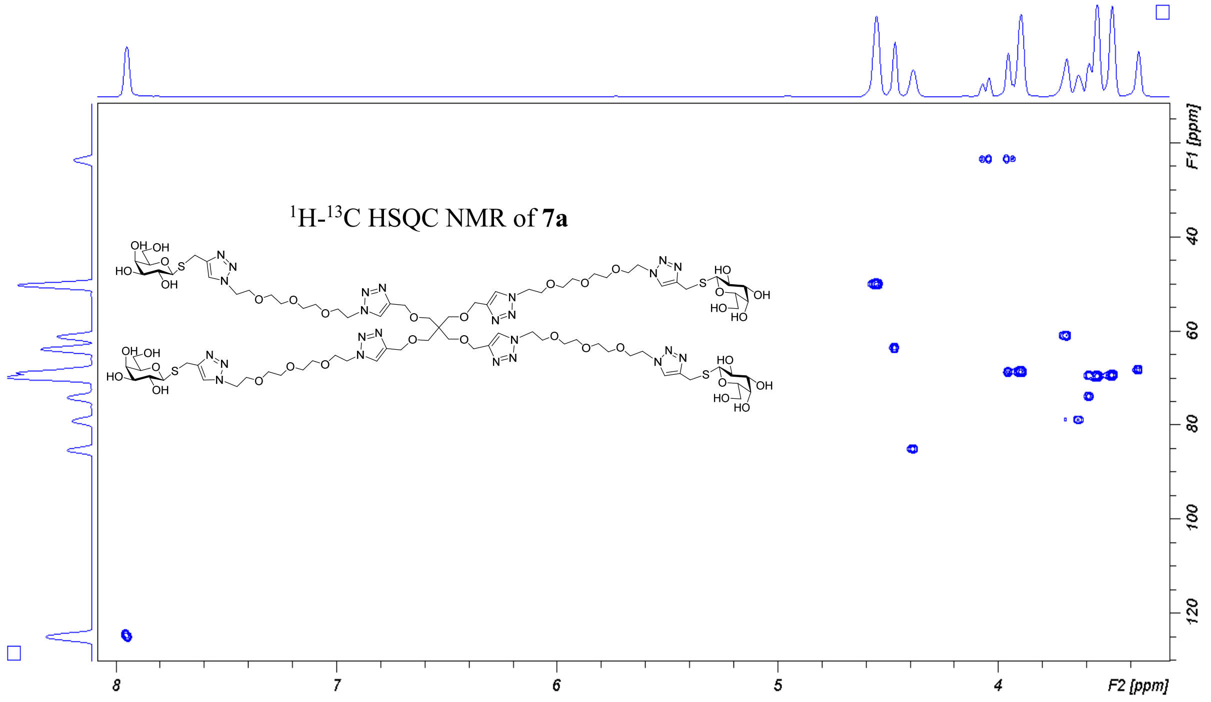

Figure S1. cont.

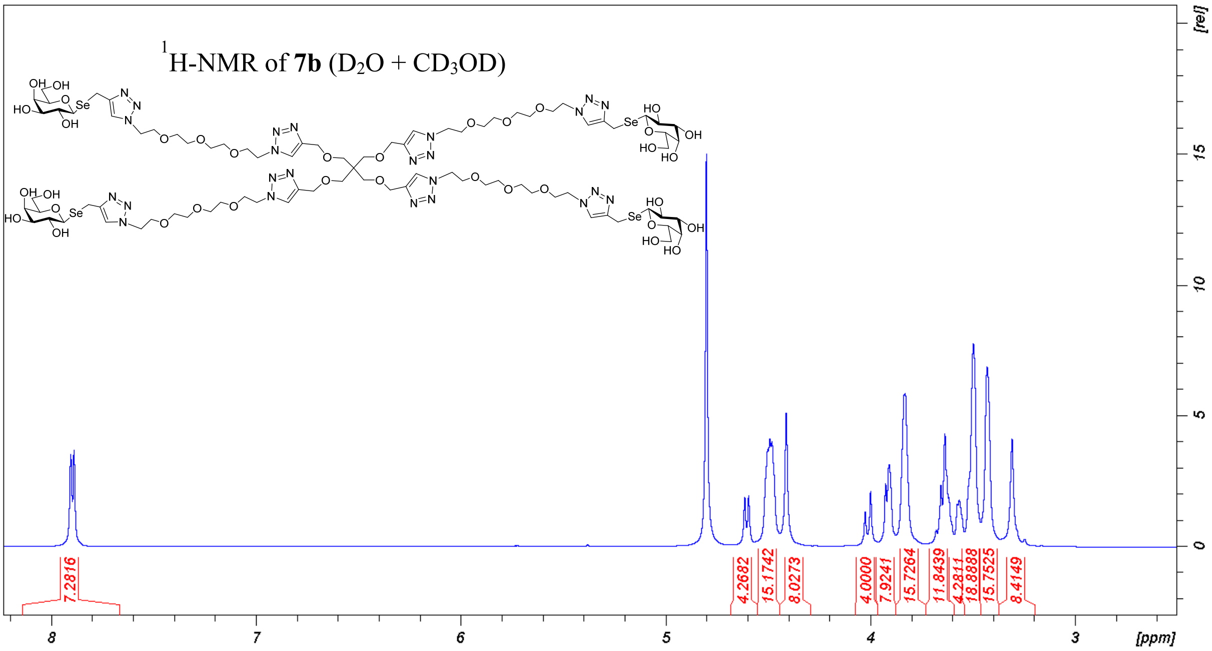

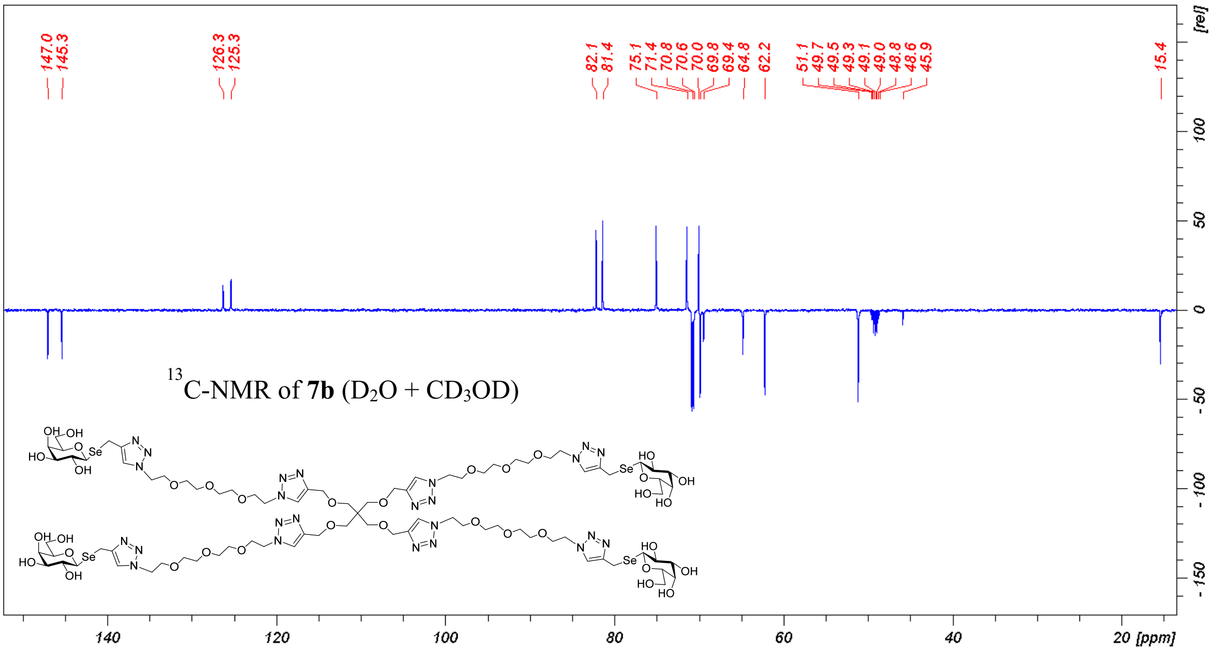

Figure S1. cont.

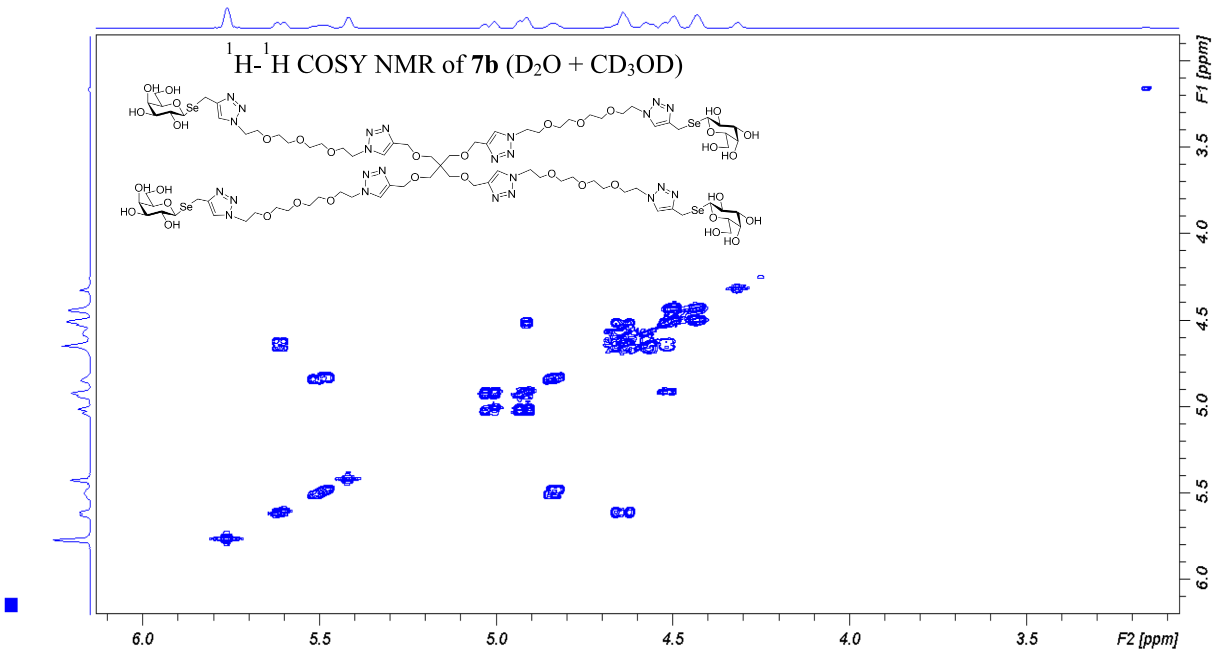

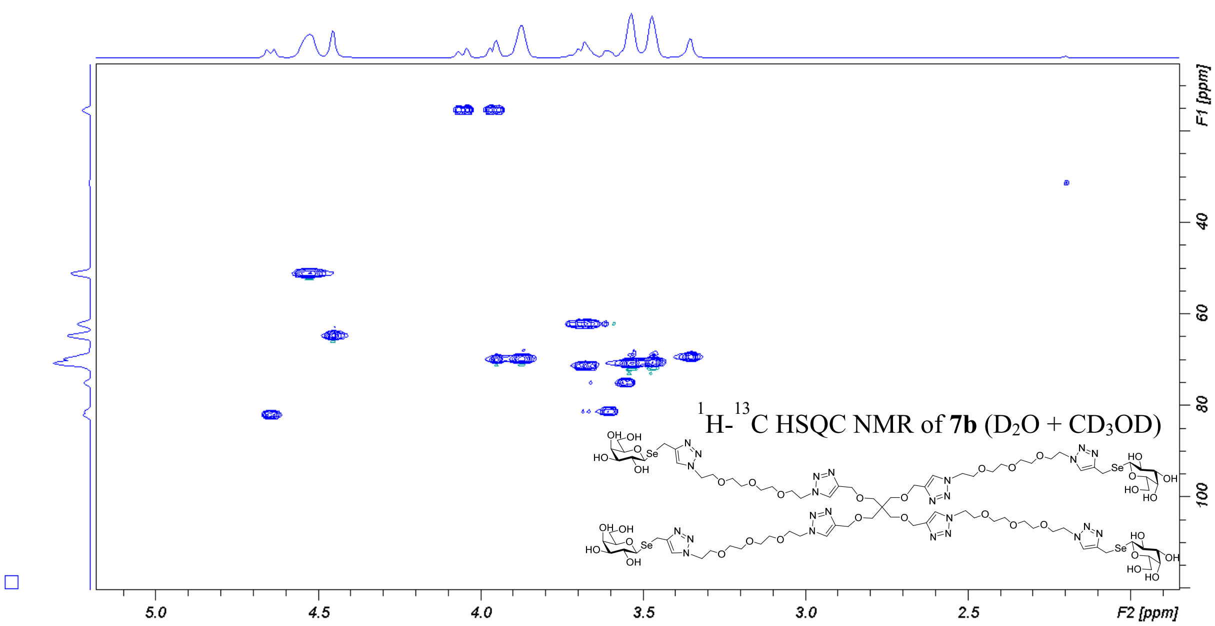

Figure S2. STD NMR spectra of **1** and **3a, 3b** in the presence of PA-IL tetramer.

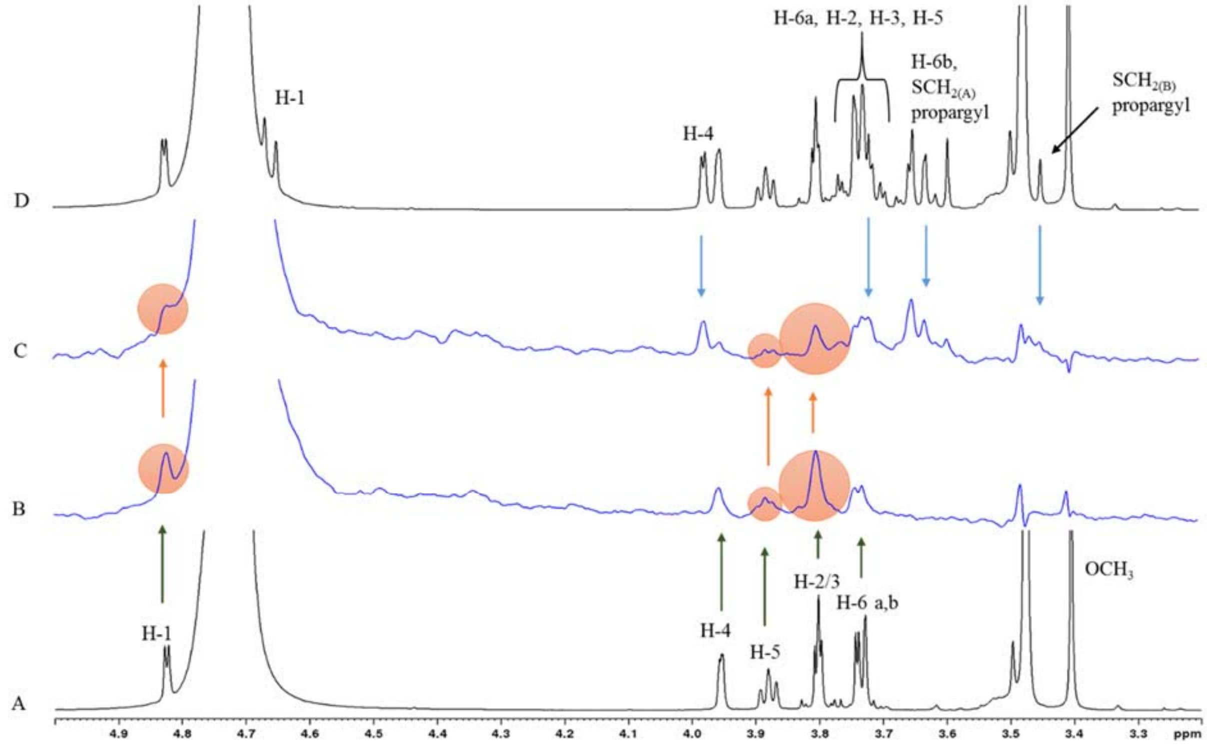

Figure S2. 500 MHz ^1H and STD NMR spectra of **1** and **3a** in the presence of 10 μM PA-IL tetramer. (A) and (B) ^1H and STD NMR spectra of **1**. (C) and (D) STD and 1H NMR spectra of the 1:1 mixture of **1** and **3a**, respectively.

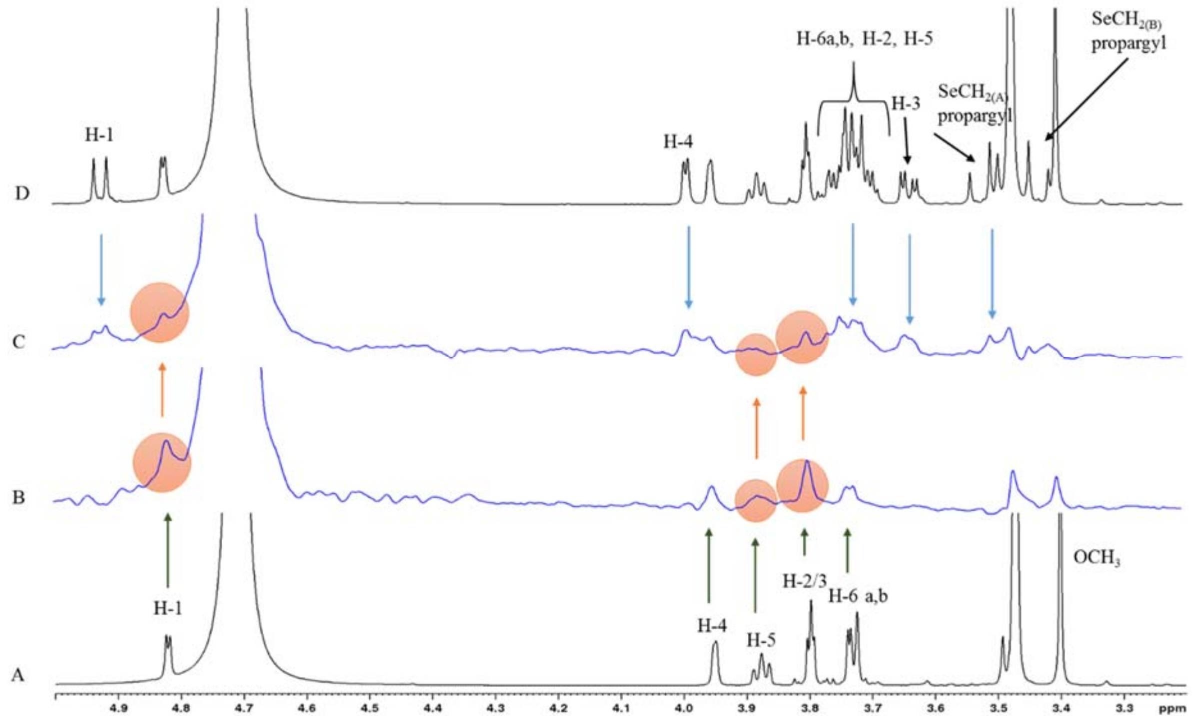

Figure S2. 500 MHz ^{1}H and STD NMR spectra of **1** and **3b** in the presence of 10 μM PA-IL tetramer. (A) and (B) ^{1}H and STD NMR spectra of **1**. (C) and (D) STD and ^{1}H NMR spectra of the 1:1 mixture of **1** and **3b**, respectively.

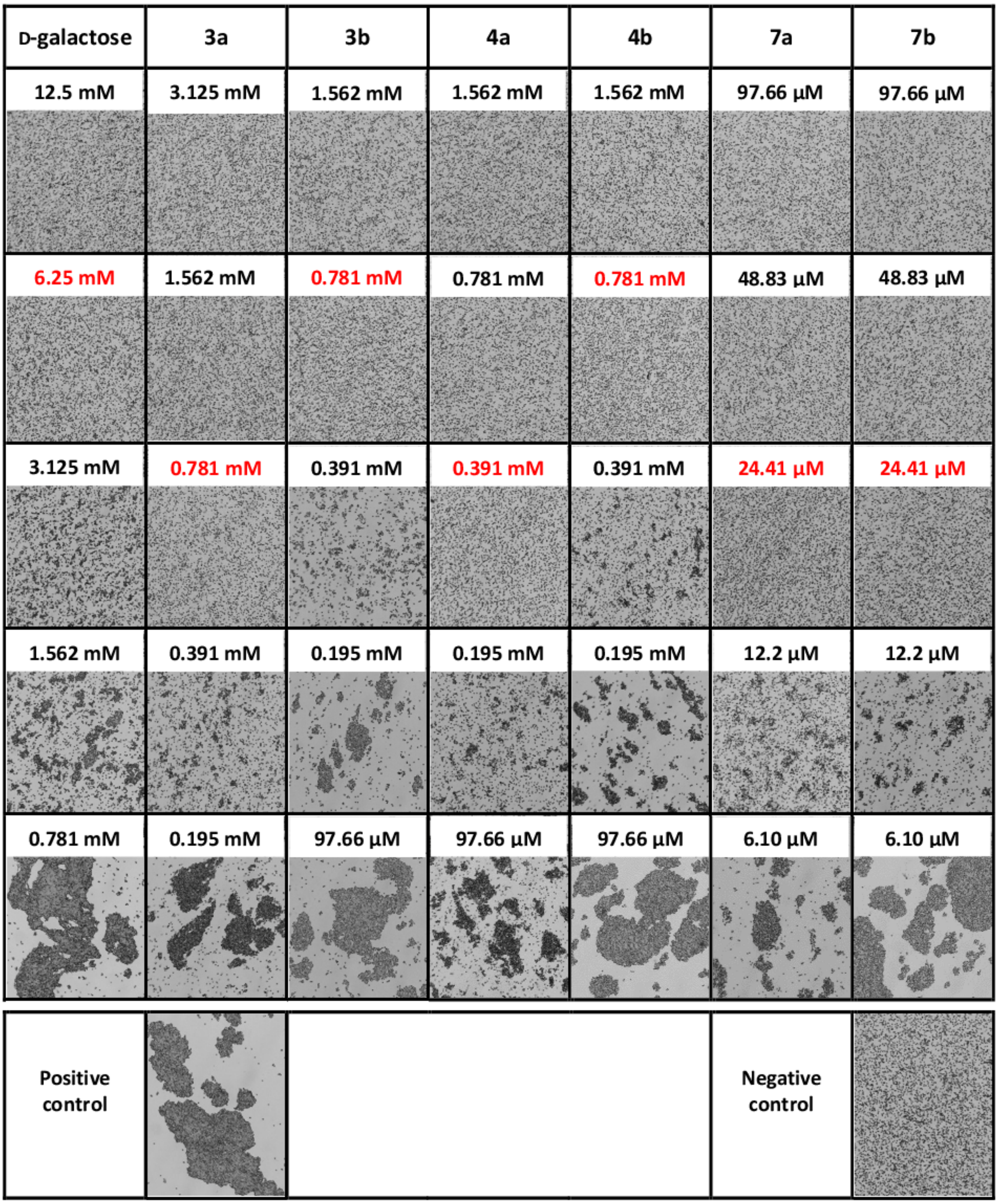

Figure S3. Influence of D-galactose, compounds **3a, 3b, 4a, 4b, 7a** and **7b** on hemagglutination caused by lectin PA-IL. Positive control: experiment without inhibitor. Negative control: experiment without lectin PA-IL. The minimal inhibitory concentration of each compound is highlighted in red.
